# A chromosome-level genome assembly provides insights into *Cornus wilsoniana* evolution, oil biosynthesis, and floral bud development

**DOI:** 10.1093/hr/uhad196

**Published:** 2023-09-29

**Authors:** Zhenxiang He, Haoyu Chao, Xinkai Zhou, Qingyang Ni, Yueming Hu, Ranran Yu, Minghuai Wang, Changzhu Li, Jingzhen Chen, Yunzhu Chen, Yong Chen, Chunyi Cui, Liangbo Zhang, Ming Chen, Dijun Chen

**Affiliations:** State Key Laboratory of Pharmaceutical Biotechnology, School of Life Sciences, Nanjing University, Nanjing 210023, China; State Key Laboratory of Pharmaceutical Biotechnology, School of Life Sciences, Nanjing University, Nanjing 210023, China; Department of Bioinformatics, College of Life Sciences, Zhejiang University, Hangzhou 310058, China; State Key Laboratory of Pharmaceutical Biotechnology, School of Life Sciences, Nanjing University, Nanjing 210023, China; Department of Bioinformatics, College of Life Sciences, Zhejiang University, Hangzhou 310058, China; Department of Bioinformatics, College of Life Sciences, Zhejiang University, Hangzhou 310058, China; State Key Laboratory of Pharmaceutical Biotechnology, School of Life Sciences, Nanjing University, Nanjing 210023, China; Forest Protection Department, Guangdong Academy of Forestry, Guangzhou 510520, China; State Key Laboratory of Utilization of Woody Oil Resource, Hunan Academy of Forestry, Changsha 410004, China; State Key Laboratory of Utilization of Woody Oil Resource, Hunan Academy of Forestry, Changsha 410004, China; State Key Laboratory of Utilization of Woody Oil Resource, Hunan Academy of Forestry, Changsha 410004, China; Xishan Forest Farm, Dazu District, Chongqing 402360, China; Longshan Forest Farm, Lechang 512221, China; State Key Laboratory of Utilization of Woody Oil Resource, Hunan Academy of Forestry, Changsha 410004, China; Hunan Horticultural Research Institute, Hunan Academy of Agricultural Sciences, Changsha 410125, China; Department of Bioinformatics, College of Life Sciences, Zhejiang University, Hangzhou 310058, China; State Key Laboratory of Pharmaceutical Biotechnology, School of Life Sciences, Nanjing University, Nanjing 210023, China

## Abstract

*Cornus wilsoniana* W. is a woody oil plant with high oil content and strong hypolipidemic effects, making it a valuable species for medicinal, landscaping, and ecological purposes in China. To advance genetic research on this species, we employed PacBio together with Hi-C data to create a draft genome assembly for *C. wilsoniana*. Based on an 11-chromosome anchored chromosome-level assembly, the estimated genome size was determined to be 843.51 Mb. The N50 contig size and N50 scaffold size were calculated to be 4.49 and 78.00 Mb, respectively. Furthermore, 30 474 protein-coding genes were annotated. Comparative genomics analysis revealed that *C. wilsoniana* diverged from its closest species ~12.46 million years ago (Mya). Furthermore, the divergence between Cornaceae and Nyssaceae occurred >62.22 Mya. We also found evidence of whole-genome duplication events and whole-genome triplication γ, occurring at ~44.90 and 115.86 Mya. We further inferred the origins of chromosomes, which sheds light on the complex evolutionary history of the karyotype of *C. wilsoniana*. Through transcriptional and metabolic analysis, we identified two *FAD2* homologous genes that may play a crucial role in controlling the oleic to linoleic acid ratio. We further investigated the correlation between metabolites and genes and identified 33 MADS-TF homologous genes that may affect flower morphology in *C. wilsoniana*. Overall, this study lays the groundwork for future research aimed at identifying the genetic basis of crucial traits in *C. wilsoniana*.

## Introduction

*Cornus wilsoniana* Wangerin (2*n* = 2*x* = 22, synonym of *Swida wilsoniana*) is a deciduous shrub belonging to the genus *Cornus* [1], and is one of the 54 perennial woody plants (https://eol.org/pages/60422/names) native to China in the northern temperate zone. The name of the *Cornus* genus is derived from the Latin word *cornu*, which translates to ‘horn’ and is a reference to the plant’s hard wood. The species epithet *wilsoniana* is a tribute to the botanist Ernest Wilson, who collected this particular species during the 1900s. Additionally, it is commonly known as Wilson’s dogwood. During spring, this particular species produces clusters of white flowers called corymbs. These flowers then develop into purplish-black berries that appear in early autumn. While the color of its flowers and stems may not be as striking as some of its more well-known relatives, its flaking, mottled bark makes it particularly attractive during the winter months, earning it the common name of ghost dogwood. In China, it is also known as the Guangpi tree. Given its year-round ornamental value, *C. wilsoniana* is a great choice for landscape design as a specimen plant.

In China, *C. wilsoniana* is an important tree species with significant ecological and economic value. The fruit of this plant is spherical, with a diameter ranging from 6.0 to 9.3 mm, and contains between 10 and 17% oil. Moreover, the sarcocarp of the fruit contains 55–59% oil [2]. The edible oil derived from *C. wilsoniana* fruit has been consumed for more than a century. This oil primarily consists of three types of fatty acid, namely palmitic acid (~15%), oleic acid (~30%), and linoleic acid (~40%) [3, 4]. Recent study has uncovered that *C. wilsoniana* possesses a potent hypolipidemic effect, which is attributed to its high concentration of polyunsaturated fatty acids, particularly linoleic acid and γ-linolenic acid [3]. Therefore, *C. wilsoniana* fruit oil can serve as a well-balanced dietary oil, not only for its hypolipidemic function but also for overcoming essential fatty acid deficiency.

Fats and oils obtained from plants constitute a crucial part of the human diet. Over the past few years, there has been growing interest in genomics research aimed at exploring the biosynthesis of oils in high-oil-bearing plants [5–12]. Compared with common vegetable oils, the oil from *C. wilsoniana* seeds has a higher content of unsaturated fatty acids than palm oil, similar to peanut, soybean, and olive oils, but lower than *Camellia* and rapeseed oils [13]. Research conducted by Liao *et al*. indicates that the saponification value of *C. wilsoniana* oil is higher than that of *Camellia* and peanut oils, but lower than soybean oil. Its peroxide value is higher than that of most other oil-bearing plants, but lower than peanut. The acid value is higher than that of most common vegetable oils, but lower than *Camellia*, soybean, and rapeseed oils [13]. Overall, the physicochemical properties of *C. wilsoniana* fruit oil demonstrates favorable performance. However, there is presently a deficiency in chromosome-level reference genome assets for *C. wilsoniana*. Consequently, the genomic underpinnings of oil biosynthesis within this species remain inadequately comprehended. Luo *et al*. have undertaken initial investigations into the pivotal *FATTY ACID DESATURASE7* (*FAD7*) gene during the fruit development process [14]. They have unveiled the dynamic patterns of gene expression but have yet to unravel the molecular regulatory mechanisms. However, in other oil-bearing plants, many transcription factors have been experimentally proven to be involved in oil biosynthesis [15–18]. For example, *WRINKLED1* (*WRI1*) can directly regulate multiple genes involved in oil biosynthesis [19] and the B3 domain transcription factor *FUSCA3* (*FUS3*) triggers the expression of various fatty acid biosynthetic genes, such as *FATTY ACID DESATURASE3* (*FAD3*) and *FATTY ACID ELONGATION*1 (*FAE1*) [20]. To fill this knowledge gap in *wilsoniana*, further genomic studies are needed, including the development of a nuclear chromosome-level reference genome using advanced genomic technologies. This will enable researchers to identify the genes involved in oil biosynthesis and better understand the molecular mechanisms underlying the production of fatty acids and oils in this important plant species.

Genome sequencing provides valuable resources for studying species evolution, identifying genetic variations, and selecting desirable traits for crop improvement [21]. To advance our understanding of *C. wilsoniana*, we utilized PacBio and Hi-C data to generate a draft genome. We then performed a systematic annotation of genomic characteristics, such as repeat sequences, non-coding RNA (ncRNA) genes, and protein-coding genes. Comparative genomics analysis revealed that *C. wilsoniana* has a closely related species, *Cornus controversa*. They are both members of Cornaceae, and they diverged 12.46 million years ago (Mya). Furthermore, the divergence between Cornaceae and Nyssaceae occurred >62.22 Mya. We also found evidence of whole-genome duplication (WGD) events and whole-genome triplication γ (WGT-γ), occurring at ~44.90 and 115.86 Mya. Moreover, we conducted an analysis of karyotype evolution, elucidating the chromosomal composition of *C. wilsoniana* and its correspondence with protochromosomes. Finally, we conducted an investigation of potential genes involved in oil biosynthesis and floral bud development in *C. wilsoniana*. Specifically, we found that *FATTY ACID DESATURASE2* (*FAD2*) homologous genes may play a crucial role in controlling the ratio of oleic to linoleic acid (O/L ratio). We further investigated the correlation between metabolites and genes and showed that 33 MADS-TF homologous genes may affect flower morphology in *C. wilsoniana*. Such information offers valuable insights into trait modification and breeding of this important plant species. Overall, the chromosome-scale assembly of the *C. wilsoniana* genome lays the foundation for future evolutionary research on the Cornaceae family and genomics research in this species. The genome data will also facilitate the identification of genes responsible for key agronomic traits, accelerating the breeding and improvement of *C. wilsoniana* as a valuable crop plant.

## Results

### Genome survey, assembly, and assessment of *Cornus wilsoniana*

**Figure 1:**
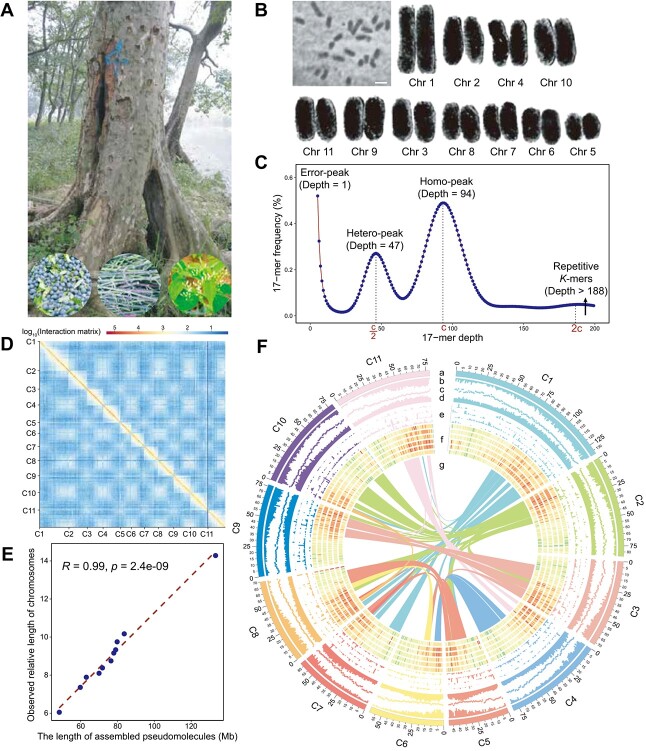
High-quality assembly and genome features of *C. wilsoniana*. **A** Photograph of *C. wilsoniana*. The three photographs at the bottom show fruits, vines, and young leaves from left to right. **B** Karyotype of *C. wilsoniana*. Bar, 1 μm. The separate chromosomes were digitally extracted for comparison. **C** The *k*-mer distribution of genome size estimation and polymorphism of *C. wilsoniana*. ‘c’ is *k*-mer depth at the homo-peak. **D** Scaffolding of 11 pseudomolecules based on chromosome conformation captured by Hi-C sequencing. Resolution, 500 kb. **E** Correlation between assembly lengths and observed physical lengths of all chromosomes. **F** Genome features depicted by using 1-Mb-wide bins across the 11 chromosomes. Track a, the 11 chromosomes. Track b, gene density (range 1–92 per 1 Mb). Track c, GC content (31–39% per 1 Mb); the orientation is outward. Track d, TE (TE) density (range 293–1281 per 1 Mb). Track e, ncRNA density (range 0–10 per 1 Mb); from the outside to the inside: miRNA, rRNA, snRNA, and tRNA. Track f, read density (range 0–5.8 per 1 Mb); the data are transformed by log_10_ and averaged from biological replicates; from the outside to the inside: leaf, bud with high yield, bud with low yield, fruit with high yield, fruit with low yield, and flower. Center g, curve lines link the syntenic regions that have been retained presumably since the WGD event.

To construct the genome of *C. wilsoniana*, fresh young leaves were harvested from Guangdong, China (Fig. 1A). The somatic cells of *C. wilsoniana* are diploid and contain 11 pairs of chromosomes, comprising 20 long metacentric chromosomes and 2 short submetacentric chromosomes (Fig. 1B and Supplementary Data Table S1). Based on *k*-mer analysis using 123× coverage Illumina short-reads, the genome size of *C. wilsoniana* was estimated to be 941.39 Mb. (Fig. 1C and Supplementary Data Table S2). The genome assembly was further improved using single-molecule real-time (SMRT) sequencing technology from PacBio. The resulting genome assembly consisted of 529 contigs, with a contig N50 of 4.49 Mb and a maximum length of 23.07 Mb, totaling 904.62 Mb (Table 1). This assembly size is consistent with the estimated genome size of 941.39 Mb based on *k*-mer analysis (Fig. 1C and Supplementary Data Table S3).

**Table 1:** Statistics for the *C. wilsoniana* genome assembly.

	**Contig level (Illumina + PacBio)**	**Scaffold level (Illumina + PacBio + Hi-C)**	**Chromosome level (Illumina + PacBio + Hi-C)**
Total number of contigs/scaffolds	529	356	11
Total length of sequences (bp)	904 624 738	904 654 138	843 505 137
Shortest sequence length (bp)	6642	6642	48 466 712
Longest sequence length (bp)	23 068 660	133 174 022	133 174 022
Contig/scaffold N50 size (bp)	4 490 562	78 005 221	78 005 221
Contig/scaffold N90 size (bp)	965 924	48 466 712	59 859 763
Total number of Ns	20	29 414	29 414
GC (%)	35.63	35.63	35.58
Number of genes	31 640	31 610	30 474

We employed the Hi-C technique to scaffold the contigs into pseudochromosomes (Supplementary Data Table S2). A total of 843.51 Mb (93.24%) of sequences were successfully anchored onto the 11 pseudochromosomes (Fig. 1D and Supplementary Data Fig. S1). There were 345 contigs, totaling 61.12 Mb, which remained unanchored to the 11 pseudochromosomes. However, our analyses showed that these 345 unanchored contigs should be regarded as redundant genomic sequences rather than contaminants originating from external sources (Supplementary Data Fig. S2). Finally, the scaffold N50 was 78.00 Mb, with a maximum chromosome length of 133.17 Mb, resulting in a high-quality scaffold-level genome assembly totaling 904.65 Mb (Table 1). We validated the assembly by comparing the physical lengths of the 11 sets of chromosomes from somatic cells with the assembly lengths, and found that they were consistent (Fig. 1E). These 11 chromosomes ranged in size from 59.86 to 133.17 Mb (Fig. 1F) and contained only 1500–5008 gaps each (Supplementary Data Table S4). The Illumina short reads mapped to the assembled *C. wilsoniana* sequences with a mapping rate of 98.83% and a coverage of 99.44%. Additionally, ~93.49% of the mRNA sequencing reads mapped to the genome sequences. We assessed assembly completeness using the Benchmarking Universal Single-Copy Orthologs (BUSCO) database, which showed that 97.71% of the core genes were complete, with only 0.9% missing in the assembled genome sequences (Supplementary Data Table S5). Collectively, these results indicate that the assembly is of high quality, with high accuracy, continuity, and genome coverage.

### Genome annotation of *Cornus wilsoniana*

#### Repeat sequences

Repetitive sequences were identified using both homology-based and *de novo* approaches, resulting in the identification of ~499.55 Mb (55.22%) of repetitive sequences (Supplementary Data Table S6). Long terminal repeat retrotransposons (LTR-RTs) were found to be the most abundant transposable elements (TEs), accounting for 48.83% of the genome, followed by DNA repeats, which accounted for only 0.79% (Supplementary Data Table S7). Analysis of copy divergence based on Kimura distance revealed that no significant recent accumulation of LTR-RTs and long interspersed nuclear elements (LINEs) was detected (Supplementary Data Fig. S3), but a concordant accumulation around the *K*_s_ peak at 0.41 was detected. Based on a substitution rate similar to that found in *Liriodendron* (1.51 × 10^−9^ substitutions/site/year) [22], we estimated that this burst of TEs occurred about 135 Mya, as calculated using the formula TE burst time = *K*_s_/(2 × nucleotide substitution rate). LTRs were found to have made the main contribution to this ancient expansion of repeat copy numbers, with an increase of up to ~1.2% at 41 units (Supplementary Data Fig. S3), indicating that the dynamics of LTR-RTs have played critical roles in the expansion of the *C. wilsoniana* genome.

#### Gene identification and annotation

Gene identification and annotation in *C. wilsoniana* was performed using a combination of *de novo*, homology-based, and RNA-seq-based predictions, resulting in a total of 31 640 protein-coding genes being predicted (Supplementary Data Fig. S4A and Supplementary Data Table S8), with 30 474 genes distributed across the 11 chromosomes (Fig. 1F). Chromosome 1 had the highest number of protein-coding genes (4943), while chromosome 5 had 1702 protein-coding genes (Supplementary Data Table S4). A total of 375 syntenic blocks were identified in the *C. wilsoniana* genome, including 8152 gene pairs (Fig. 1F). The average coding sequence length of 1158 bp in *C. wilsoniana* was similar to that of five other related plant species investigated (Supplementary Data Fig. S4B), while the average gene length of 6739 bp was greater (Supplementary Data Table S9). Of the predicted genes, 29 852 (94.30%) were assigned functions based on homologies to annotated proteins in the Swiss-Prot and NR databases, and 90.60% of the genes contained conserved protein domains based on functional annotation using InterProScan. More than half (53.80%) of the genes in the *C. wilsoniana* genome were successfully annotated with Gene Ontology (GO) terms, indicating their functional categories. Additionally, the Kyoto Encyclopedia of Genes and Genomes (KEGG) pathway database was used to map 77.70% of the genes to known plant biological pathways (Supplementary Data Table S10). A total of 21 783 genes were simultaneously annotated in either of these databases (Supplementary Data Fig. S4C). In addition to the protein-coding gene annotations, ncRNA genes were also annotated, including 880 miRNAs, 727 tRNAs, 676 rRNAs, and 1335 snRNAs (Supplementary Data Table S11).

### Comparative genomics analysis

**Figure 2:**
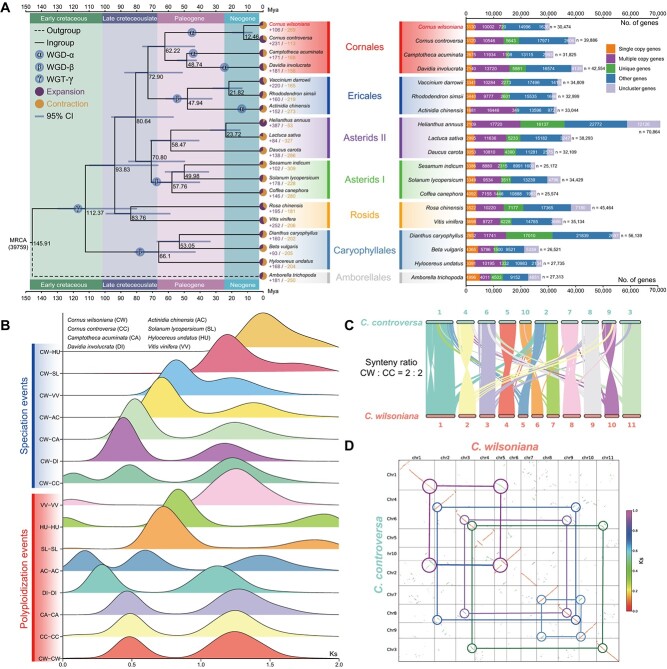
Phylogenetic analysis of the *C. wilsoniana* genome. **A** Phylogenetic tree for *C. wilsoniana* and 18 other plants. Genes of *C. wilsoniana* and other sequenced genomes are divided into five classes and the direct numbers (*n*) are shown on the right with bar charts. Gene family expansions or contractions are indicated in a solid circle; corresponding proportions among the total changes are shown in the pie charts. Estimated divergence time (Mya) is indicated at each node with the 95% confidence interval (CI). The ball with embedded text represents recent WGD or WGT-γ events. *Cornus wilsoniana* is highlighted. **B***K*_s_ values revealed two WGD events during the evolution of *C. wilsoniana*. **C** Collinear relationships between *C. wilsoniana* and *C. controversa*. All banded lines in the background indicate syntenic blocks between genomes spanning >20 genes. **D** Syntenic block dotplot between *C. wilsoniana* and *C. controversa*. A syntenic depth ratio of 2:2 is shown using circular and solid lines.

We conducted a comparative genomics analysis involving 19 species, encompassing a range of taxonomic orders including Cornales (*Cornus wilsoniana*, *C. controversa*, *Camptotheca acuminata*, and *Davidia involucrata*), Ericales (*Vaccinium darrowii*, *Rhododendron simsii*, and *Actinidia chinensis*), asterids I (*Sesamum indicum*, *Solanum lycopersicum*, and *Coffea canephora*), asterids II (*Helianthus annuus*, *Lactuca sativa*, and *Daucus carota*), rosids (*Vitis vinifera* and *Rosa chinensis*), Caryophyllales (*Dianthus caryophyllus*, *Beta vulgaris*, and *Hylocereus undatus*), and Amborellales (*Amborella trichopoda*). This comprehensive selection of species was employed to investigate the evolutionary patterns within the genome of *C. wilsoniana*. A total of 39 759 gene families were analyzed, revealing 6475 common gene families, and 254 were specific gene families in the *C. wilsoniana* genome (Supplementary Data Table S12). For each species, the orthogroup gene statistics were computed, and *C. wilsoniana* had 3130 (10.27%) single-copy genes and 10 002 (32.82%) multiple-copy genes (Fig. 2A and Supplementary Data Table S13). Cluster analysis of gene families for selected plant species, including *C. wilsoniana*, *C. controversa*, *Actinidia chinensis*, *Amborella trichopoda*, *Beta vulgaris*, *Camptotheca acuminata*, and *Coffea canephora*, revealed both conserved and specific gene families (Supplementary Data Fig. S5A). A total of 720 specific genes were identified (Supplementary Data Fig. S5B). GO and KEGG enrichments were used to analyze the function of these specific genes in *C. wilsoniana*. The GO analysis (adjusted *P*-value <.05) showed that the specific genes in *C. wilsoniana* were involved in hydrolase activity and carbohydrate metabolic process, suggesting their potential importance in specific catalytic hydrolysis reactions (Supplementary Data Fig. S5C and Supplementary Data Table S14). The KEGG analysis results showed that the specific genes were enriched (adjusted *P*-value <.05) in cyanoamino acid metabolism and terpenoid backbone biosynthesis, indicating their potential involvement in the functions of disease and pest resistance, immune suppression, and lipid reduction (Supplementary Data Fig. S5D and Supplementary Data Table S14) [23].

We performed a phylogenetic analysis by identifying 101 single-copy gene families among the 19 species, which revealed that *C. wilsoniana* has a closely related species, *C. controversa* (Supplementary Data Fig. S6). They are both members of Cornaceae, and they diverged 12.46 Mya (Fig. 2A). We subsequently identified 269 contracted and 106 expanded gene families, encompassing 1168 and 1105 genes, respectively (Supplementary Data Fig. S5B). The expanded genes were found to be enriched in GO terms related to signal transduction, ADP binding, and signaling receptor activity (Supplementary Data Fig. S5E and Supplementary Data Table S15). Furthermore, our investigation of the KEGG pathways revealed that the expanded gene families were notably enriched in several processes, including the MAPK signaling pathway and stilbenoid, diarylheptanoid, and gingerol biosynthesis (Supplementary Data Fig. S5F and Supplementary Data Table S15). These findings suggest that the expanded genes may have played a role in the plant’s response to pathogen attacks. In addition, our findings revealed that *C. wilsoniana* was phylogenetically closely related to Nyssaceae species, specifically *Camptotheca acuminata* and *Davidia involucrata* (Fig. 2A). The divergence between Cornaceae and Nyssaceae occurred >62.22 Mya. All four of these species belong to the order Cornales. This order shares a sister relationship with the order Ericales, which includes *Vaccinium darrowii*, *Rhododendron simsii*, and *Actinidia chinensis* (Fig. 2A). The divergence time between these two orders is estimated to have occurred around 72.90 Mya (Fig. 2A). Overall, based on the evolutionary analysis of the 19 species, we have constructed a relatively comprehensive and well-defined species phylogeny route for *C. wilsoniana*. Additionally, compared with other species, we have shown that *C. wilsoniana* underwent its final divergence to become a distinct species during the mid-Neogene period. Therefore, *C. wilsoniana* holds promise as a prospective candidate for investigating late-stage species evolution in further studies.

In order to gain insights into species divergence and WGD events, we performed whole-genome collinearity analysis involving *C. wilsoniana* and seven other species (*C. controversa*, *Camptotheca acuminata*, *Davidia involucrata*, *Actinidia chinensis*, *Solanum lycopersicum*, *Hylocerus undatus*, and *Vitis vinifera*). A total of 204 207 collinear genes pairs and 10 512 synthetic blocks were identified (Supplementary Data Table S16). The WGD and speciation events in *C. wilsoniana* were inferred using paralogous and orthologous pairs of *K*_s_ distribution, revealing peaks at ~0.48 and 1.25 (Fig. 2B). Previous studies have unveiled a characteristic pattern of triplicate sets of syntenic gene blocks in the *Vitis vinifera* genome, which is attributed to the occurrence of WGT-γ events [24]. These triplicated genome structures are believed to have originated from WGT-γ events estimated to have taken place around 117 Mya [25]. Utilizing the formula divergence time = *K*_s_/2*r*, we can deduce that *C. wilsoniana* experienced WGD events at ~44.90 and 115.86 Mya during the Early Cretaceous and Paleogene periods (Supplementary Data Table S16), respectively. Of these, the ancient WGD event (around 115.86 Mya) corresponds to the WGT-γ event (Fig. 2B), which is shared among eudicots [25]. Furthermore, *C. wilsoniana* underwent a more recent WGD event (around 44.90 Mya) subsequent to its divergence from the Nyssaceae family (Fig. 2A and B). Remarkably, this recent WGD event is also observed in its closest relative, *C. controversa*, and is similarly evident in *Camptotheca acuminata* of the Nyssaceae family, which predates the WGD occurrence in *Davidia involucrata* (Fig. 2B). Consequently, we hypothesize that this recent WGD event is shared within the Cornaceae family. In addition to the triplication event, we can also observe significant speciation events that are, on the whole, consistent with the divergent histories in (Fig. 2A and B). However, there are instances where the divergence times of *C. wilsoniana–Solanum lycopersicum* (CW − SL, *K*_s_ = 1.19) and *C. wilsoniana–Vitis vinifera* (CW − VV, *K*_s_ = 0.82) contrast with the divergence times of Cornales–rosids and Cornales–asterids, as depicted in Fig. 2A and B. We attribute this discrepancy to potential biases introduced by utilizing single-copy genes and collinear gene pairs for species divergence inference. Therefore, when specifically investigating the divergence times between two species, employing collinear gene pairs for species divergence inference is considered a more reliable approach.

In the aforementioned study, we observed a close phylogenetic relationship and shared WGD and WGT-γ events between *C. wilsoniana* and *C. controversa*. As a result, we conducted a further comprehensive genome-wide collinearity analysis on these two species, revealing a substantial degree of chromosomal collinearity at the whole-genome level (Fig. 2C). Analyzing their homologous genes, a distinct synteny ratio of 2:2 could be clearly observed (Fig. 2D). Through the comparison of syntenic gene blocks forming three *K*_s_ peaks, it was noted that the most recent peak (*K*_s_ = 0.09) contained the least number of collinear gene pairs (Fig. 2B). This implies that they retain a wide variety of similar chromosomal features during their final divergence, which is consistent with the large collinear segments shown in Fig. 2C.

**Figure 3:**
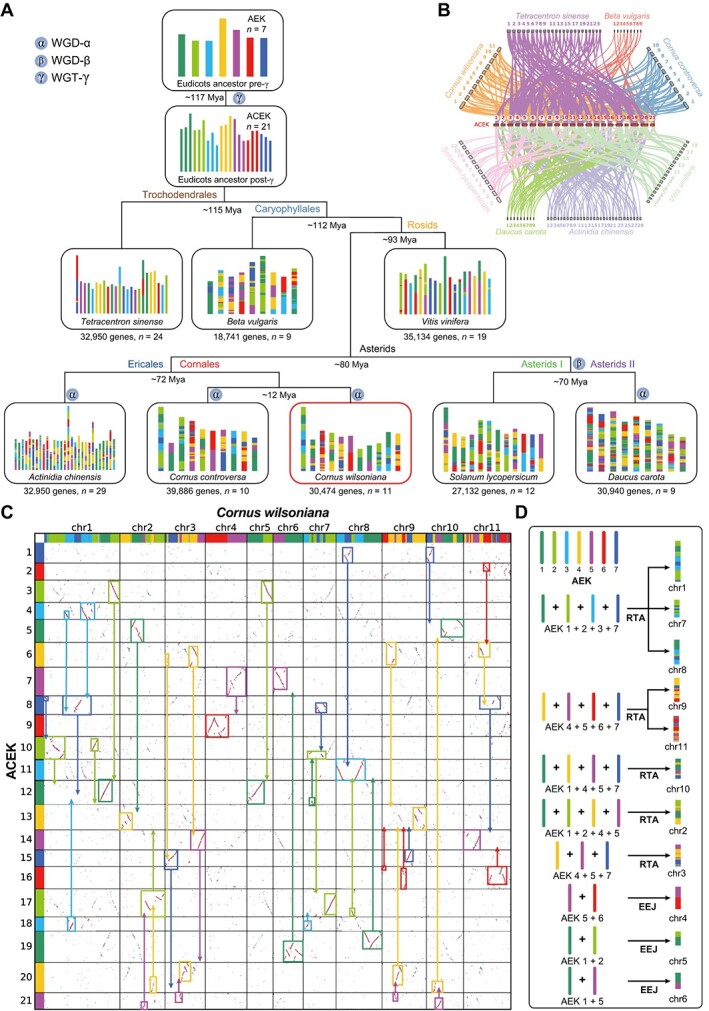
Evolutionary scenario of *C. wilsoniana* from the AEK. **A** Modern genomes are illustrated at the bottom, the different colors reflecting the origin from the seven ancestral chromosomes from the *n* = 7 AEK (top). **B** Collinear relationships between the ACEK and eight specific species. **C** Syntenic block dotplot between *C. wilsoniana* and ACEK. Reassembly of chromosomes is displayed using rectangular and solid arrows. **D** Ideograph of chromosome origin in *C. wilsoniana*.

### Karyotype evolution of *Cornus wilsoniana*

Karyotype evolution represents chromosomal changes from the ancestral genome to the modern genome. This information is critical for reconstructing ancestral karyotypes and tracing how modern species evolved step by step [26]. To assess the paleohistory of *C. wilsoniana*, we conducted an analysis of karyotype evolution across eight species (*Tetracentron sinense*, *Beta vulgaris*, *Vitis vinifera*, *Actinidia chinensis*, *C. controversa*, *C. wilsoniana*, *Solanum lycopersicum*, *Daucus carota*). We reconstructed the ancestral eudicot karyotype (AEK) with *Tetracentron sinense*, which is one of four early-diverging lineages of eudicots (Fig. 3A). Following the occurrence of the WGT-γ, the AEK evolved into the ancestral core eudicot karyotype (ACEK), featuring 21 protochromosomes (Fig. 3A). Subsequent core eudicot species then underwent fusion and fission events involving these 21 protochromosomes to generate distinct chromosomal numbers and compositions. Accordingly, we identified syntenic gene blocks shared between the ACEK and eight specific species for assessing species karyotypes (Fig. 3B).

Then, we reconstructed the karyotype evolutionary trajectory of representative species based on the phylogenetic relationships and syntenic gene blocks of the eight species (Fig. 3A). From this analysis, it becomes apparent that *Tetracentron sinense* and *Vitis vinifera* have retained relatively intact protochromosomes. As species diversified and experienced WGD events, the composition of protochromosomes in *C. wilsoniana* became notably intricate. Nonetheless, we can still infer the origins of different segments within each chromosome of *C. wilsoniana* (Fig. 3C). Furthermore, a clear 1:2 synteny ratio is evident between the ACEK and *C. wilsoniana*, reaffirming the occurrence of another WGD event in *C. wilsoniana* post-WGT-γ (Fig. 3C).

Given the considerable degree of divergence in *C. wilsoniana*, detailing its karyotypic evolutionary history becomes a complex task. However, by establishing connections between *C. wilsoniana* and the AEK through syntenic gene blocks with the ACEK, and employing mechanisms like reciprocally translocated chromosome arms (RTAs), nested chromosome fusion (NCF), and end–end joining (EEJ), we can infer the origins of chromosomes (Fig. 3D). For instance, chr1, chr7, and chr8 are thought to have formed via RTA from the AEK’s 1st, 2nd, 3rd, and 7th protochromosomes, while chr9 and chr11 arose through RTA involving the AEK’s 4th, 5th, 6th, and 7th protochromosomes. Additionally, chr4, chr5, and chr6 are the result of the fusion of two distinct AEK protochromosomes by EEJ (Fig. 3D). We believe that as the number of species genomes continues to expand, future research endeavors will enable us to unveil a comprehensive account of the intricate details of karyotype evolution.

### Identification of key genes involved in oil biosynthesis

To uncover the key genes responsible for oil biosynthesis in *C. wilsoniana*, a comparative genome analysis was conducted. The results showed that *C. wilsoniana* possessed 1663 genes involved in acyl lipid synthesis, a comparable number to soybean (1695 genes), but greater than that of non-oilseed plants such as *Arabidopsis* (1290 genes). *Cornus wilsoniana* and soybean exhibited a notable enrichment of lipid gene functional groups compared with *Arabidopsis* (Supplementary Data Table S17). While there were slight variations in the number of genes in certain categories of lipid synthesis genes, *C. wilsoniana* had a greater number of genes involved in plastidial fatty acid synthesis, lipid signaling, and triacylglycerol (TAG) synthesis.

**Figure 4:**
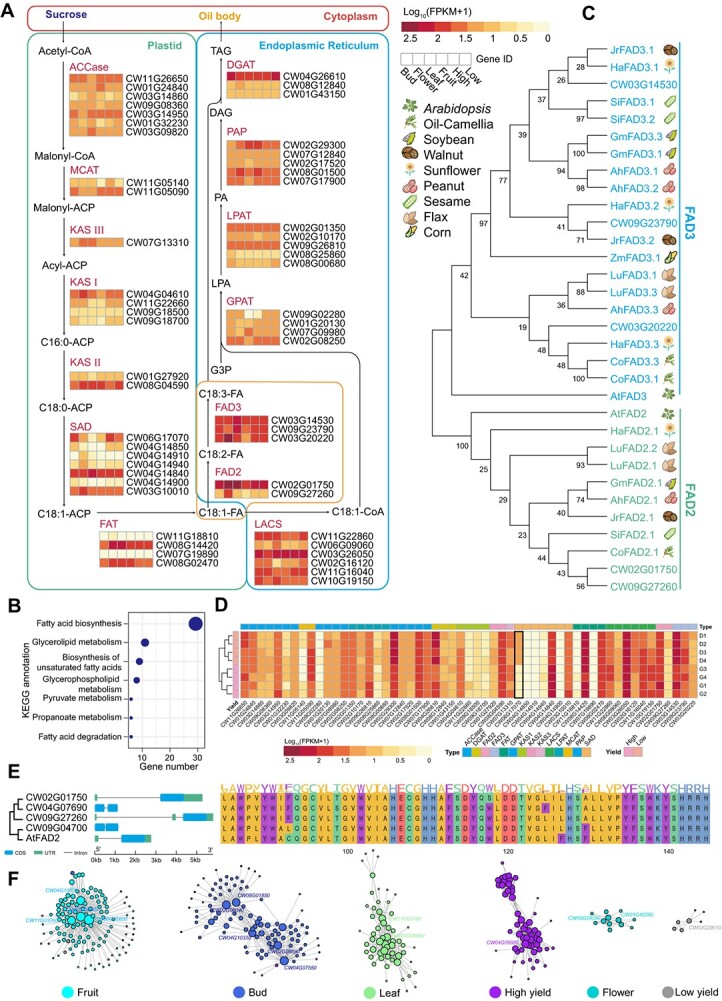
Genes involved in oil biosynthesis. **A** Simplified representation of the oil biosynthetic pathway. Expression levels were estimated by FPKM for each gene obtained by sequencing RNA samples from bud, flower, fruit, and leaf for high- and low-yield genotypes. **B** KEGG pathway annotation of *C. wilsoniana* putative oil biosynthetic genes. **C** Phylogenetic tree of candidate genes encoding FAD2 and FAD3 in 10 species. Values beside the branches represents maximum likelihood estimation (MLE) bootstrap for that branch. A cartoon illustrating species type is at the right of the phylogenetic tree. **D** Heat map of putative oil biosynthetic gene expression level in fruits of high- and low-yield genotypes. **E** Comparative analysis of DNA structure and protein domain PF00487 of four homologous *FAD2* genes in *C. wilsoniana* and AtFAD2. **F** Co-expression regulatory network constructed based on WGCNA. Node size represents the degree of connectivity, and transcription factors are marked around nodes.

To explore the genes associated with fatty acid and TAG biosynthesis, we manually examined the *C. wilsoniana* proteome and searched it against the UniProt Reference Clusters (UniRef) database [27], leading to the identification of 80 candidate genes. Among them, 55 showed expression signals by RNA-seq data from bud, flower, and leaf tissues in *C. wilsoniana* (Supplementary Data Table S18). These genes form a complete oil biosynthesis pathway, enriching the KEGG pathway (ko00061) of fatty acid biosynthesis (Fig. 4A and B). Understanding the expression diversity of these metabolic genes is crucial for improving the quality parameters of oils, particularly in achieving an O/L ratio. *FAD2*, which encodes δ-12 oleic acid desaturase and controls the high oleate trait [28], showed high expression in the late stage of blooming but low expression after seed desiccation (Fig. 4A). The expression levels of crucial enzymes involved in the TAG pathway exhibited variations across distinct developmental stages (Fig. 4A). Comparing *FAD2* (*CW02G01750* and *CW09G27260*) and *FAD3* (*CW03G14530*, *CW09G23790*, and *CW03G20220*) genes with known FAD protein genes from other crops, it was found that the FAD proteins from *C. wilsoniana* shared a close evolutionary relationship with those from sunflower [29], walnut [30], and oil *Camellia* [31], which have a high proportion of unsaturated acids (Fig. 4C). These FAD proteins contained two identical domains (PF00487 and PF11960) (Supplementary Data Fig. S7), indicating a similar function in regulating the O/L ratio. Furthermore, among the 55 genes involved in TAG biosynthesis, *CW06G17070* genes with significant differences [log_2_(fold change) = 2.85, adjusted *P*-value = 1.46e−6] in gene expression were identified between high- and low-yield fruit samples (Fig. 4D and Supplementary Data Table S19).

According to previous research, only a single *FAD2* gene has been identified in *Arabidopsis* (accession number L26296) [32]. However, in other oil crops at least one additional gene has been identified. For instance, two *FAD2* genes have been characterized in soybean (accession numbers L43920 and L43921) and olive (accession numbers AY733076 and AY733077) [33, 34], three genes in sunflower (accession numbers AF251842, AF251843, and AF251844) and *Camelina* (accession numbers HQ008320, HQ008321, and HQ008322) [35, 36], and four to six different *FAD2* genes among *Brassica* species [37]. Similarly, in *C. wilsoniana*, using the *AtFAD2* gene (*AT3G12120*) as a query, a total of four significantly expressed homologous *FAD2* genes were identified (identity >70%, *e*-value <1*e*−5, FPKM >1) (FPKM, fragments per kilobase of transcript per million mapped fragments). The presence of multiple copies of *FAD2* genes might suggest potential coordination in regulating the O/L ratio. Additionally, based on DNA sequence analysis (Fig. 4E), *CW02G01750* and *CW09G27260* share similar gene structural features with the known *AtFAD2* gene, including a single large intron in the 5′-untranslated region (UTR) and a single coding sequence. On the other hand, *CW09G04700* and *CW04G07690* lack UTR regions but have coding sequences of similar lengths to *AtFAD2*. In terms of protein sequence structure, the PF00487 domain is associated with fatty acid desaturase, and the amino acid composition of the four *FAD2* proteins in *C. wilsoniana* is highly similar to the PF00487 domain present in known *AtFAD2* (Fig. 4E). Furthermore, we observed relative conservation of amino acid types at each position (Fig. 4E), which lays the foundation for future gene engineering efforts. Finally, for a comprehensive assessment of key genes involved in oil biosynthesis, we employed weighted correlation network analysis (WGCNA) to identify four hub transcription factors (*CW02G05600*, *CW04G10500*, *CW08G05800*, *CW11G03760*) in the fruit (Fig. 4F and Supplementary Data Fig. S8). We also identified downstream genes co-expressed with these transcription factors at *P*-value <1e−5 and GS > 0.9 (Supplementary Data Table S20). We believe that these key genes will provide a fundamental basis for breeding research related to TAG biosynthesis in *C. wilsoniana*.

### Transcription and metabolism in developing floral buds

**Figure 5:**
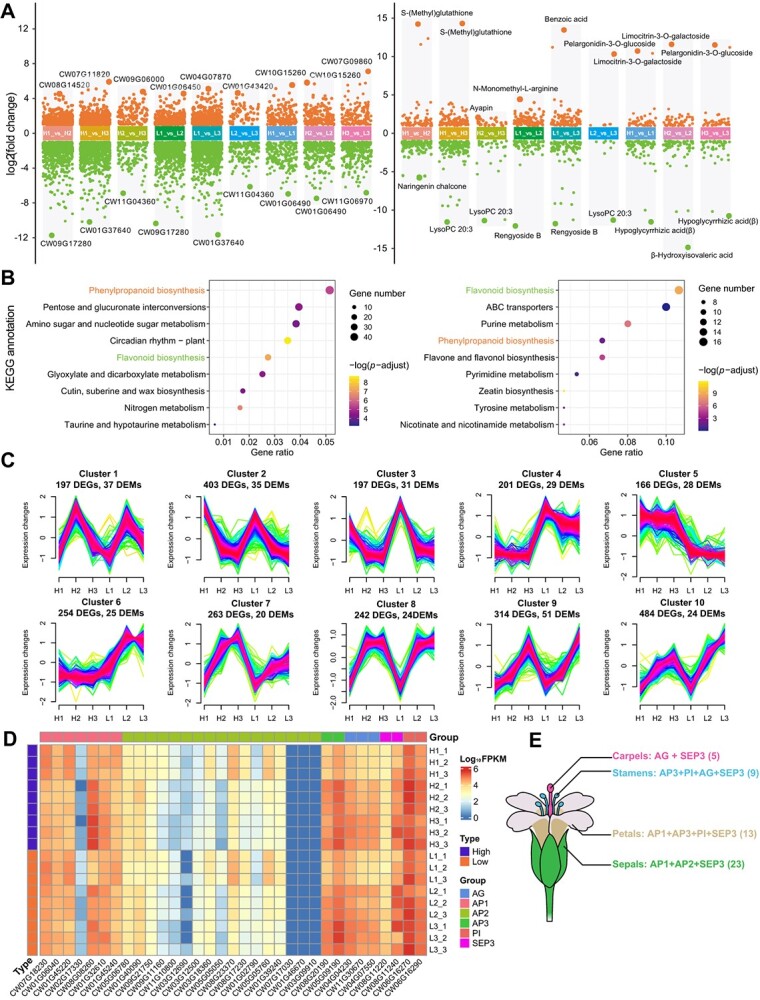
Genes and metabolites involved in the developing floral bud. **A** Scatter plot of differentially expressed genes (DEGs, left) and differentially expressed metabolites (DEMs, right). Dots represent upregulated or downregulated genes and metabolites. Genes and metabolites with the most significant differences are highlighted on each bar chart. **B** KEGG pathway annotation of *C. wilsoniana* putative floral bud development genes and metabolites. Adjusted *P*-value <.05. **C** Trend analysis identified 10 distinct patterns of DEG and DEM expression. The *x* axis represents three developmental stages for high- and low-yield traits, while the *y* axis represents log_2_-transformed, normalized intensity ratios in each stage. **D** Heat map of MADS-TF gene expression in floral bud development. Floral buds at three developmental stages, namely the calyx differentiation stage (H1 and L1), the pistil differentiation stage (H2 and L2), and the stamen differentiation stage (H3 and L3), from two genotypes with high yield (H) and low yield (L). **E** MADS-TF gene co-regulatory landscape for floral organs. The number of MADS-TF genes is shown in parentheses.

Transcription and metabolism during floral bud development are critical processes for plant blooming [38]. Here, transcriptomics and metabolomics techniques were employed to investigate changes during floral bud development. Principal component analysis (PCA) of the three biological replicates demonstrated high data reproducibility (Supplementary Data Figs S9 and S10). Throughout various developmental stages of the floral buds, namely the calyx differentiation stage (H1 and L1), the pistil differentiation stage (H2 and L2), and the stamen differentiation stage (H3 and L3), a set of 2721 genes and 424 metabolites displayed significant differential expression (Fig. 5A, Supplementary Data Tables S21 and S22). Interestingly, some of the most significant differentially expressed genes (DEGs) and differentially expressed metabolites (DEMs) were detected for both high- (H) and low-yield (L) traits during the same developmental stage, such as *CW09G17280* in the H1 versus H2 and L1 versus L2 comparisons and *CW11G04360* and lysoPC (20:3) in the H2 versus H3 and L2 versus L3 comparisons, indicating their potential impact on flower morphology. These DEGs and DEMs were also found to be involved in the same KEGG pathways, such as phenylpropanoid biosynthesis and flavonoid biosynthesis (Fig. 5B), which are related to color and aroma formation in *C. wilsoniana* [39]. To further investigate the correlation between metabolites and genes, we conducted a trend analysis of DEGs and DEMs. We identified a total of 10 clusters (Fig. 5C, Supplementary Data Fig. S11, and Supplementary Data Table S23) that collectively exhibit consistent patterns of change in both DEGs and DEMs across high- and low-yield traits. Moreover, within these 10 clusters, we performed a correlation analysis on DEGs and DEMs enriched in the phenylpropanoid biosynthesis pathway (ko00940) and flavonoid biosynthesis pathway (ko00941). This analysis revealed several strongly correlated gene–metabolite pairs, such as *CW05G15020*–caffeoylshikimic acid in cluster 2, *CW01G00140*–coniferin in cluster 4, and *CW08G05520*–phloretin in cluster 9 (Supplementary Data Fig. S12).

Furthermore, floral bud differentiation is not only a process of tissue development and function improvement but also a nutrient accumulation process. Soluble nutrients, such as sucrose and amino acids, are transported from vegetative organs to floral buds and subsequently converted into insoluble polymer compounds like starch, protein, and fatty acids. This gradual process culminates in the formation of the primary components of flowers and prepares them for further blooming [40]. During the flowering stage, various plant organs, particularly floral organs, harbor a multitude of MADS-TF genes. In the case of *C. wilsoniana*, we identified 33 MADS-TF genes (Supplementary Data Table S24), which include six key transcription factors, *AP1* (*APETALA1*), *AP2*, *AP3*, *AG* (*AGAMOUS*), *PI* (*PISTILLATA*), and *SEP3* (*SEPALLATA3*), directly associated with flower development [41]. Among these, all transcription factors except *CW07G17030*, *CW01G46670*, and *CW03G09910* from the *AP2* group exhibited significant expression levels during flower development. Previous research indicates that MADS-TF genes collaborate to orchestrate diverse morphologies of floral organs [41]. Therefore, we reconstructed a transcription factor co-regulatory landscape governing the development of sepals, petals, stamens, and carpels in *C. wilsoniana* (Fig. 5E). Overall, these findings provide valuable insights into the development of floral buds and offer genetic resources for molecular breeding in *C. wilsoniana*.

## Discussion

By generating the draft genome of *C. wilsoniana*, our study has furnished a valuable resource for comprehending the genetic foundation and evolution of oil biosynthesis and floral bud development within the Cornaceae family. In this study, we generated a chromosome-scale assembly of the *C. wilsoniana* genome using PacBio and Hi-C data. A phylogenetic analysis identifies *C. controversa* as the closest species to *C. wilsoniana*, sharing a common divergence from the Cornaceae family ~12.46 Mya. Notably, *C. wilsoniana* is closely related to Nyssaceae species. Our study outlines divergence times between various species, constructing a well-defined species phylogeny route for *C. wilsoniana* and suggesting its potential for investigating late-stage species evolution. WGD and speciation events in *C. wilsoniana* are inferred through *K*_s_ distribution analysis, indicating ancient and recent WGD events around 115.86 and 44.90 Mya, respectively. This recent WGD event is shared within the Cornaceae family and plays a role in the divergence of *C. wilsoniana* and its closest relatives. We employ mechanisms like RTA, NCF, and EEJ to infer the origins of chromosomes, which sheds light on the complex evolutionary history of the karyotype of *C. wilsoniana*.

The high oil content of *C. wilsoniana* fruit has attracted increasing attention in recent years due to its strong potential hypolipidemic effect. In this study, we identified key genes and pathways involved in oil biosynthesis in *C. wilsoniana*. Our results suggested that *C. wilsoniana* shares common pathways with other high-oil-bearing plants, such as palmitic acid, oleic acid, and linoleic acid biosynthesis. However, we also found potential genes that may contribute to the high O/L ratio of *C. wilsoniana* fruit, such as the *FAD2* gene family members [42]. These findings provide a foundation for further studies on the genetic basis of oil biosynthesis and medicinal value in *C. wilsoniana* and other high-oil-bearing plants. Besides, we also investigated the genetic basis of floral bud development in *C. wilsoniana*. Our analysis identified several key MADS-TFs involved in floral bud development, including *AP1* [43, 44], and *SEP* [45] homologous genes. We found that these genes are highly conserved across different plant species and are essential for floral organ development. Insights into the molecular mechanisms governing floral bud development in *C. wilsoniana* were gained through our study, which could pave the way for breeding new cultivars with superior floral traits.

Overall, our study provides a valuable resource for comprehending the evolution and genetic basis of crucial traits in *C. wilsoniana*. The chromosome-scale assembly of the genome in *C. wilsoniana* can facilitate the discovery of genes and pathways linked to significant traits and may expedite the breeding of new cultivars with better floral characteristics and oil content. Furthermore, our research has implications for plant evolution and the genetic mechanisms of complex traits in other plant species. Further functional investigations of the crucial genes highlighted in this study are necessary to enhance our understanding of the genetic foundation of crucial traits in *C. wilsoniana* and other plants with high oil content.

## Materials and methods

### Plant materials

The biological samples utilized in this study were sourced from *C. wilsoniana* tissue materials, which were procured from the cultivation field of the seed resource bank at Guangdong Academy of Forestry (113°38′0″, 23°20′0″). The study encompassed floral buds at distinct developmental stages: the calyx differentiation stage (H1 and L1), the pistil differentiation stage (H2 and L2), and the stamen differentiation stage (H3 and L3). These stages were examined in two genotypes characterized by high yield (H) and low yield (L). A total of three independent biological replicates were collected for each sample. Furthermore, flower samples were harvested at the initial flowering stage (F1) and the blooming stage (F2), independently, with two biological replicates for each stage. Fresh young leaves (Le) were collected prior to the onset of the initial flowering stage and were represented by two biological replicates. Seeds from two distinct genotypes, distinguished as high yield (G) and low yield (D), were harvested post-desiccation. For each sample, four biological replicates were collected. All gathered materials were rapidly frozen using liquid nitrogen and subsequently stored at −80°C until they underwent RNA sequencing and metabolite extraction procedures.

### DNA extraction and sequencing

To extract DNA from fresh young leaves of *C. wilsoniana*, the cetyltrimethylammonium bromide (CTAB) method was employed due to its proven ability to yield high-quality genomic DNA. Subsequently, the obtained DNA underwent fragmentation and was used to create libraries containing inserts of >30 kb. Our specific strategy involved selecting DNA fragments with inserts of 20 kb, which were subsequently subjected to sequencing on the PacBio Sequel platform. In total, six SMRT cells were utilized for the sequencing process. The raw PacBio subreads underwent processing through the SMRT Link pipeline, resulting in an assembly of ~6.12 million subreads. These subreads exhibited an average length of 22.9 kb, achieving a genome coverage of 150× and generating a total sequencing output of 120 Gb. To address potential assembly errors, an Illumina DNA-seq library was prepared using the same DNA sample. Paired-end sequencing with 350-bp reads was conducted on the Illumina HiSeq X Ten platform, yielding a dataset of 116 Gb and a genome coverage of 120×. For the Hi-C experiment, ~3 g of fresh young leaf tissue was pulverized under liquid nitrogen to create a fine powder. This material underwent a series of steps including chromatin extraction, enzymatic digestion, DNA ligation, purification, and fragmentation, culminating in the generation of a Hi-C library. The meticulously prepared library was sequenced using the Illumina HiSeq X Ten platform, significantly contributing to the achievement of a chromosome-level genome assembly. Detailed information about the dataset can be found in Supplementary Data Table S2.

### Estimation of genome size

To determine the genome size of *C. wilsoniana*, we employed the conventional *k*-mer counting technique. This involved analyzing the occurrence of *k*-mers within the clean Illumina short reads (with a size of 17 bp) using Jellyfish [46]. Our assessment of sequencing depth hinged on identifying the peak value in the distribution frequency curve of *k*-mer occurrences, specifically focusing on homo-peak characteristics. By analyzing a total of 89 452 735 820 *k*-mers, we pinpointed the *k*-mer depth corresponding to the homo-peak, which was found to be 94. Acknowledging that a *k*-mer depth of 1 could be attributed to errors, we accounted for this by considering an error rate of 1.08%. This error rate was then employed to refine our genome size calculations. The initial estimate of the genome size was derived from the ratio of the total *k*-mer count to the homo-peak depth. To further improve accuracy, we factored in the error rate, resulting in the revised estimate: (*k*-mer count)/(homo-peak depth) × (1 − error rate). Based on these calculations, the estimated genome size of *C. wilsoniana* was determined to be ~951.62 Mb. However, after applying the correction, the revised genome size was recalculated to be ~941.39 Mb (as detailed in Supplementary Data Table S3). Additionally, GCE v1.0.2 [47] was employed to estimate both the heterozygous ratio and the proportion of repeat sequences.

### Genome *de novo* assembly

For *de novo* assembly, we utilized Falcon v2.0 in conjunction with PacBio clean reads [48]. The assembly process incorporated specific parameters seed_coverage = 30 and length_cutoff_pr = 4 K, which facilitated optimal results. Subsequently, the initial draft assembly underwent refinement steps. Arrow v2.2 was applied to polish the assembly [49], utilizing PacBio clean reads. Following this, further polishing was executed using Pilon v1.22 [50], employing Illumina clean reads. To eliminate any redundant contigs and enhance the assembly’s conciseness, we incorporated Purge_Haplotigs into our pipeline [51]. Ensuring the integrity of the assembly and uniform sequencing coverage was pivotal. To achieve this, we performed realignment of continuous long-read subreads onto the final assembly using Minimap2 v2.5 with default parameters [52]. In our pursuit of evaluating the quality of the assembled genome, we conducted a comprehensive analysis. We employed BUSCO v3.0.2 [53], employing the Embryophyta_odb10 database, and adhered to the default parameters. This assessment allowed us to gauge the completeness of gene content within the assembled genome.

### Chromosome-scale assembly using Hi-C

To integrate the structural information provided by Hi-C data, we performed a series of steps to refine our scaffold assembly. Initially, the Hi-C clean data were aligned to the scaffold assembly using BWA-mem v0.7.17 [54]. We exercised stringent selection criteria, specifically utilizing read pairs where both reads aligned to contigs and were positioned within 500 bp of a restriction site. Furthermore, only uniquely mapped read pairs that represented valid interaction paired-end reads were considered. These meticulously chosen read pairs were subsequently employed to construct pseudochromosome sequences, enhancing the accuracy of our genome representation. The process of assembling, ordering, and orienting the scaffolds to form the coherent structure of the 11 chromosomes specific to *C. wilsoniana* was undertaken using LACHESIS in conjunction with Juicebox v1.8.8 [55, 56]. To visually present the interaction patterns within the Hi-C data, we generated a Hi-C matrix heat map. This heat map, offering insights into chromosomal interactions, was created using HiCExplorer 3.7.2 [57], with a resolution of 500 kb.

### Genome annotation

#### Repeat annotation

Our strategy for repeat annotation harnessed a dual approach, integrating homology alignment and *de novo* search techniques to comprehensively identify whole-genome repeats. For the detection of tandem repeats, we employed the TRF tool (http://tandem.bu.edu/trf/trf.html) through *ab initio* prediction [58]. To ensure an inclusive evaluation, we leveraged the Repbase database (http://www.girinst.org/repbase, version 15.02) [59], a well-recognized resource for homolog prediction. RepeatMasker (http://www.repeatmasker.org, version 3.3.0) software, along with its in-house scripts (RepeatProteinMask), was applied with default parameters to facilitate the extraction of repeat regions [59]. A *de novo* repetitive element database was built using LTR_FINDER (https://github.com/xzhub/LTR_Finder, version 1.05), RepeatScout (http://www.repeatmasker.org/, version 1.0.5), and RepeatModeler (http://www.repeatmasker.org/RepeatModeler.html, version 1.0.5) with default parameters. In the construction of the raw TE library, preference was given to repeat sequences >100 bp in length and possessing a gap containing <5% ‘N’ nucleotides. To unmask DNA-level repeats, we amalgamated the Repbase and *de novo* TE libraries to create a customized composite library [59]. Subsequently, this library underwent a curation process using uclust [60], resulting in the establishment of a non-redundant library. This refined library was then furnished to RepeatMasker for comprehensive repeat annotation [61].

#### Structure annotation

The comprehensive annotation of gene models within the genome involved a well-rounded approach that encompassed *ab initio* prediction, homology-based prediction, and RNA-seq-assisted methods. To enhance the accuracy of homolog prediction, a curated set of homologous protein sequences was procured from NCBI (https://www.ncbi.nlm.nih.gov/). Employing TblastN v2.2.26 with an *e*-value threshold of ≤1*e*−5 [62], these protein sequences were aligned against the genome, thereby establishing sequence homology. To refine this alignment and decipher gene structures within the identified protein regions, the GeneWise v2.4.1 tool was employed [63]. The *ab initio* gene prediction process encompassed the utilization of multiple tools to predict coding genes within the repeat-masked genome. Augustus v3.2.3 [64], Geneid v1.4 [65], GENSCAN v1.0 [66], GlimmerHMM v3.04 [67], and SNAP [68] were harnessed in this endeavor, collectively contributing to the comprehensive prediction of coding regions. Expanding the gene annotation pipeline, RNA-seq data from young leaves played a pivotal role. These transcriptome reads were aligned to the *C. wilsoniana* genome utilizing TopHat v2.0.11 69, with parameters carefully configured as -p 10 -N 3 --read-edit-dist 3 -m 1 -r 0 --coverage-search --microexon-search [69]. This alignment process led to the identification of exon regions and splice sites, serving as the foundation for genome-based transcript assembly. Subsequent assembly was accomplished using Cufflinks v2.2.1 with default parameters [70]. To harmonize these diverse gene predictions and generate a concise reference gene set, EvidenceModeler v1.1.1 was applied [71]. This tool facilitated the merging of genes predicted through the three methods. Importantly, PASA terminal exon support was incorporated, with the inclusion of masked TEs as inputs into gene prediction. The ensuing step involved the informed selection of specific gene families for manual curation. This pivotal task was undertaken by domain experts, ensuring a refined and accurate gene annotation outcome.

To generate a non-redundant reference gene set, we merged genes predicted by the three methods using EvidenceModeler v1.1.1 with PASA terminal exon support, including masked TEs as input into gene prediction. Subsequently, the selection of specific families for further manual curation was carried out by relevant experts.

#### Functional annotations

For the comprehensive assignment of gene functions, a multi-pronged approach was undertaken, integrating sequence alignment, motif/domain annotation, and ontology assignment. To initiate the process, protein sequences were meticulously aligned to the Swiss-Prot database [72]. This alignment was orchestrated using Diamond with a stringent *e*-value threshold of ≤1*e*−5 [73], ensuring only high-confidence matches were considered. The outcomes of this alignment played a foundational role in unraveling the functional annotations. Motifs and domains, pivotal indicators of protein functionality, were meticulously elucidated through the application of InterProScan v5.31 [74]. This tool enabled a comprehensive search across an array of publicly available databases, encompassing ProDom [75], PRINTS [76], Pfam [77], SMART [78], PANTHER [79], and PROSITE [80]. This thorough exploration ensured that a wide spectrum of potential motifs and domains were identified. The assignment of GO IDs, vital for contextualizing gene functions, was executed with precision. The basis for these assignments was the corresponding entries within the InterPro database [74], thereby establishing a structured link between sequences and functional annotations. To enhance the accuracy of protein function predictions, we employed a strategy that involved the transfer of annotations from the closest BLAST hit in the NR database. Specifically, hits with *e*-values ≤1*e*−5 were chosen for this purpose. The NR database (https://ftp.ncbi.nlm.nih.gov/blast/db/FASTA/) served as a comprehensive resource for extracting relevant annotations. Furthermore, we harnessed the power of the KEGG pathway database to map the gene set to relevant pathways [81]. By identifying the best match for each gene within the KEGG pathways, we enriched our understanding of the functional context in which each gene operates.

#### Non-coding RNA annotation

The prediction of transfer RNAs (tRNAs) was accomplished using the specialized tRNAscan-SE program (RRID:SCR 010835, version 1.4) [82]. This tool, tailored for tRNA prediction, played a pivotal role in uncovering the presence and locations of tRNA genes within the genome. Given the high degree of conservation exhibited by ribosomal RNAs (rRNAs), a comparative approach was adopted. We utilized the rRNA sequence of a closely related species as a reference template. Employing BLAST with an *e*-value threshold of 1*e*−5 [83], rRNA sequences within our target genome were predicted, leveraging sequence similarities with the reference. To comprehensively identify other non-coding RNA classes, including microRNAs (miRNAs) and small nuclear RNAs (snRNAs), a meticulous search strategy was deployed. The Rfam database (http://infernal.janelia.org/) [84], renowned for housing a diverse collection of non-coding RNA families, was explored using the Infernal software (RRID:SCR 011809, version 1.1). This software, tailor-made for the detection of non-coding RNAs, enabled the systematic identification of these functionally significant RNA molecules.

### Comparative analysis of genomes between species

#### Gene family and phylogenetic analysis

To gain comprehensive insights into the protein-coding sequences present in the *C. wilsoniana* genome, we embarked on a comparative genomics endeavor encompassing 18 diverse species spanning a range of taxonomic orders. These species included representatives from Cornales (*C. controversa* [85], *Camptotheca acuminata* [86], and *Davidia involucrata* [87]), Ericales (*Vaccinium darrowii* [88], *Rhododendron simsii* [89], and *Actinidia chinensis* [90]), asterids I (*Sesamum indicum* [5], *Solanum lycopersicum* [91], and *Coffea canephora* [92]), asterids II (*Helianthus annuus* [6], *Lactuca sativa* [93], and *Daucus carota* [94]), rosids (*Vitis vinifera* [95] and *Rosa chinensis* [96]), Caryophyllales (*Dianthus caryophyllus* [97], *Beta vulgaris* [98], and *Hylocereus undatus* [99]), and Amborellales (*Amborella trichopoda* [100]). In our pursuit of representing genes with alternative splicing variants, we selected the transcript housing the longest coding sequence (coding sequence) to serve as a representative for each gene. To gauge the similarities between sequence pairs, we harnessed BLASTp with a stringent *e*-value cutoff of 1*e*−5, facilitating an in-depth comparison. The OrthoMCL v2.0.9 toolkit proved invaluable in our exploration of gene family membership [101]. Utilizing a comprehensive approach, we performed all-against-all protein sequence similarity searches, adopting Markov chain clustering to discern the intricate relationships. From the OrthoMCL results, 101 single-copy orthologous genes were extracted. Subsequently, an alignment was crafted using Muscle v3.8.31 [102] with default parameters. Gblocks v0.91b was employed to judiciously eliminate ambiguously aligned positions [103].

To unravel the intricate branches of evolution, we harnessed RAxML v8.2.12 to construct a phylogenetic tree [104]. The datasets from *Amborella trichopoda* served as the outgroup for this analysis. A robustness assessment was conducted through the execution of 1000 bootstrap analyses. Intrigued by the temporal aspects of evolution, we adopted a Bayesian relaxed molecular clock method. This method, facilitated through MCMCTree in PAML [105], enabled us to estimate species divergence time. We relied on the calibration time from the TimeTree database [106], specifically centered around the divergence between *C. wilsoniana* and *Amborella trichopoda*. To shed light on gene family dynamics, we embraced CAFE v5.0.0 [107], conceptualizing gene family evolution as a stochastic birth and death process. Parameters –p 0.05 -r 1000 were specified for this analysis. Furthermore, we delved into the intricate nuances of gene family evolution. This involved a meticulous analysis of changes in gene family sizes across the phylogenetic tree. To discern functional implications, we subjected expanded gene families to GO and KEGG enrichment analysis. These analyses were orchestrated using clusterProfiler 4.0 [108].

#### Whole-genome duplication analysis and identification

For the identification of WGDs, we harnessed DIAMOND v0.9.29.130 [73], employing it to pinpoint gene pairs displaying substantial sequence similarities (with an *e*-value <1*e*−5). Subsequently, to ensure the robustness of our findings, we invoked a stringent *C* score threshold. Specifically, gene pairs with a *C* score >0.5 were retained, with this filtering process facilitated by JCVI software [109]. To uncover the spatial relationships of these similar gene pairs, we adopted MCScanX [110], a powerful tool designed to discern adjacency patterns on chromosomes and establish collinear gene blocks. The outcomes of this analysis allowed us to identify instances of conserved genomic organization, shedding light on the evolutionary trajectory of duplicated gene blocks. The collinearity patterns were graphically depicted through collinearity plots, which were meticulously drawn using JCVI software [109]. To delve into the intricacies of WGD events within the *C. wilsoniana* genome, we turned to wgd v1.1.0 software [111]. This specialized tool, tailor-made for WGD analysis, facilitated the identification of instances where large-scale duplication events have shaped the genomic landscape. To gain insights into the evolutionary implications of these WGD events, we engaged the codeml utility within PAML [105]. This multifaceted approach enabled us to glean a deeper understanding of the impact of WGD on gene evolution in *C. wilsoniana*.

#### Karyotype evolution

The comprehensive suite of analyses was orchestrated through the utilization of WGDI [112], an essential tool in unraveling the intricate patterns within genomic data. This encompassed a series of methodical steps, each contributing to the holistic understanding of genomic organization and evolution. To initiate the exploration of chromosome structure within *Tetracentron sinense*, we employed WGDI in conjunction with the -d parameter. This approach facilitated the creation of dot plots, enabling us to visually discern the underlying chromosome architecture. Subsequently, the -ak parameter of WGDI was invoked to unravel the AEK and ACEK components inferred by *Tetracentron sinense*. These inferred elements offered critical insights into the ancestral genomic organization. For other species under consideration, we delved into the realm of inter-genomic relationships. Utilizing the -icl parameter, we unearthed collinear genes between the ACEK and specific species, offering a comparative perspective on genomic organization. The -bi parameter was subsequently harnessed to identify collinear genes between these collinear blocks. To streamline the analysis and facilitate the identification of collinear fragments, we judiciously integrated these collinear blocks using the -bi parameter. This integration enabled us to pinpoint cohesive genomic regions representing shared evolutionary history. The analysis continued with a stringent filtering process. Utilizing the -c parameter, we systematically excluded blocks that may have stemmed from more ancient evolutionary events, ensuring the focus remained on relevant patterns of evolution. To validate the reasonableness of the remaining blocks, the -bk parameter was employed. This critical validation step ensured that the collinear blocks under consideration exhibited a coherent evolutionary trajectory. For the mapping of the AEK, the -km parameter was employed, culminating in the elucidation of the AEK’s intricate mapping across different species. To visually encapsulate the inter-species collinearity, we resorted to the JCVI software [109], which generated inter-species collinearity plots. These plots distilled complex genomic relationships into coherent visuals, offering a snapshot of the shared evolutionary dynamics.

#### Genes involved in oil biosynthesis and analysis

To elucidate the genetic components governing oil biosynthesis, we initiated a meticulous retrieval and comparative analysis of relevant genes. The foundation of this analysis lay in the comprehensive aralip database (http://aralip.plantbiology.msu.edu/downloads), housing genes associated with oil biosynthesis in *Arabidopsis*. In a bid to uncover orthologous genes in soybean and *C. wilsoniana*, we initiated a sequence similarity search. Deploying the DIAMOND tool with stringent criteria (*e*-value ≤1*e*−5, alignment identity ≥40%, and alignment coverage ≥50%), we established connections between genes. These connections were subsequently mapped back to categories within the aralip database, culminating in the quantification of genes associated with oil biosynthesis. A parallel investigation was undertaken, involving the searching of 80 *C. wilsoniana* genes linked to oil biosynthesis. This search was executed against the UniProt Reference Clusters (UniRef) database (https://www.uniprot.org/help/downloads) using the *C. wilsoniana* proteome. The DIAMOND tool, with identical parameters to the previous analysis, enabled the identification of homologous genes. These homologs were further examined for expression signals, with 55 genes displaying signals in at least one sample. Leveraging these genes, we embarked on the design of pathways for TAG biosynthesis.

To comprehend the evolutionary relationships within fatty acid desaturase (FAD) proteins, we devised a sophisticated strategy. Commencing with the retrieval of FAD proteins across eight oil-bearing crops (corn, flax, sesame, peanut, sunflower, walnut, soybean, and oil *Camellia*) and *Arabidopsis*, we used the *C. wilsoniana* FAD proteins as the basis. This dataset was employed for phylogenetic tree construction via the maximum likelihood method, realized through MEGA 11 [113]. The accuracy of the analysis was bolstered through the computation of 1000 bootstrap replicates, while values <50% were judiciously excluded. The structural aspects of FAD proteins were explicated through the retrieval of protein domains from InterPro (https://www.ebi.ac.uk/interpro/search). GeneDoc 2.7 (http://nrbsc.org/gfx/genedoc) facilitated a comprehensive visualization of the gene structure. For a nuanced understanding of structural domain nucleotide composition, the ggmsa tool was employed [114]. In the realm of gene co-expression networks, we harnessed the capabilities of the R package WGCNA [115]. This tool was pivotal in constructing intricate networks that unveiled the interplay between genes. For the visualization of these networks, igraph (https://igraph.org) was seamlessly integrated, ensuring a coherent representation of intricate relationships.

#### Transcription analysis

Samples were collected and subjected to RNA isolation and purification using the pBIzol kit (BIOFLUX, Hangzhou Bori Technology, Hangzhou, China). Subsequently, cDNA libraries were constructed and subjected to sequencing. Quality assessment of the extracted RNA samples was a critical step in the pipeline. This was realized through a combination of techniques. A NanoDrop ultraviolet spectrophotometer (Thermo, Waltham, MA, USA) facilitated the quantification of RNA, while the integrity of the samples was assessed using the Bioanalyzer 2100 System (Agilent, Santa Clara, CA, USA). This comprehensive evaluation assured the suitability of the RNA for downstream analysis. For the construction of cDNA libraries, we integrated ~3 μg of the pristine RNA samples. These libraries, meticulously prepared, were the foundation for subsequent sequencing endeavors. Leveraging the capabilities of the Illumina HiSeq 4000 platform, we embarked on high-throughput sequencing, generating paired-end reads spanning 150 bp. This sequencing was entrusted to the expertise of Allwegene Biotechnology Co., Ltd. (Beijing, China). The raw sequencing reads, in FASTQ format, underwent stringent preprocessing. Trimmomatic v0.39 was invoked to adeptly remove adapters and sequences of low quality [116]. This quality-centric processing ensured that the downstream analysis would be based on high-fidelity sequences. To establish an intricate alignment with the reference genome of *C. wilsoniana*, we leveraged the STAR v2.7.10b tool [117]. This alignment process facilitated the accurate mapping of the processed reads, paving the way for a granular exploration of gene expression patterns. To quantify gene expression levels, we turned to RSEM 1.3.3 [118], a tool renowned for its precision in calculating FPKM. This metric served as a robust indicator of gene expression dynamics. To identify differentially expressed genes, a critical aspect of our analysis, we deployed DESeq2 v1.34 [119]. With a keen focus on statistical significance and biological relevance, this tool facilitated the distinction of genes exhibiting altered expression profiles. Our criteria for differential expression encompassed a *P*-value threshold of ≤.05 and an absolute log_2_(fold change) value of >1.

#### Metabolome analysis

Floral bud samples at calyx differentiation stage, pistil differentiation stage, and stamen differentiation stage were used for their metabolome analysis. Before ultraperformance liquid chromatography–mass spectrometry (MS)/MS analysis, the samples were first freeze-dried and then ground into a fine powder [120, 121]. The mass spectrum data were processed using Analyst 1.6.3. The metabolites present in the samples were identified and quantitatively and qualitatively analyzed by MS using the local metabolic database [122, 123]. To conduct the PCA and partial least-squares discriminant analysis (PLS-DA), we used the ropls v1.26.4 [124] package to identify significantly changed metabolites, with threshold of |log_2_(fold change)| ≥ 1，VIP ≥ 1, and *P*-value ≤.05.

## Acknowledgements

The authors would like to express their gratitude to the Hunan Academy of Forestry, Guangdong Academy of Forestry, Longshan Forest Farm in Lechang City of Guangdong Province, Xishan Forest Farm in Dazu District of Chongqing Municipality, and Yangkou State-owned Forest Farm in Fujian Province for their efforts in selecting superior resources and collecting, preserving, and propagating *C. wilsoniana* materials. The authors would also like to thank Yicun Chen from the Subtropical Forestry Research Institute of the Chinese Academy of Forestry for providing the *Litsea cubeba* genome data. The authors are also grateful to the members of Ming Chen’s laboratory for helpful discussions and valuable comments. This work was supported by the National Natural Science Foundation of China (31770767, 32070656, 32070677 and 32270709); Foundation of State Key Laboratory of Utilization of Woody Oil Resource (GZKF202201); Hunan Province Key Research and Development Program Project (2018NK2044); Jiangsu Collaborative Innovation Center for Modern Crop Production and Collaborative Innovation Center for Modern Crop Production co-sponsored by province and ministry.

## Author contributions

Z.H., L.Z., M.C., and D.C. conceived the study and provided data. H.C. performed the data analyses, visualization, and manuscript writing. H.C., Q.N., Y.H., and R.Y. performed the data interpretation. X.Z. established the databases. Z.H., L.Z., M.C., and D.C. supervised the work and provided scientific advice. M.W., C.L., J.C., Yunzhu C., Yong C., and C.C. contributed significantly to the selection of superior resources, collecting, preserving, and propagating *C. wilsoniana* materials. All authors contributed to writing and editing the final manuscript.

## Data availability

The raw genome and transcriptome sequencing data of *C. wilsoniana* have been deposited in the Genome Sequence Archive (https://ngdc.cncb.ac.cn/gsa: accession no. CRA010376) [125]. The raw metabolome data has been deposited in OMIX (https://ngdc.cncb.ac.cn/omix/releaseList: accession no. OMIX003453), and the chromosome-level genome assembly and annotation files have been deposited in GWH (https://ngdc.cncb.ac.cn/gwh: accession no. GWHCAYR00000000) at the National Genomics Data Center, China National Center for Bioinformation/Beijing Institute of Genomics, Chinese Academy of Sciences [126]. The reference genome assembly and annotation files of *C. wilsoniana* at contig and scaffold level are also available for free download on our website (https://biobigdata.nju.edu.cn/cornus).

## Conflict of interest

The authors declare that they have no conflict of interests.

## Supplementary Material

Supplementary_Tables_uhad196

SupFigs_uhad196
