# Supplementary material for: A chromosome-level genome assembly provides insights into Cornus wilsoniana evolution, oil biosynthesis, and floral bud development
Source: Hortic Res. 2023 Sep 29;10(11):uhad196. doi: 10.1093/hr/uhad196 (PMC10673659; doi:10.1093/hr/uhad196)
Supplement: SupFigs_uhad196 [file supfigs_uhad196.pdf]

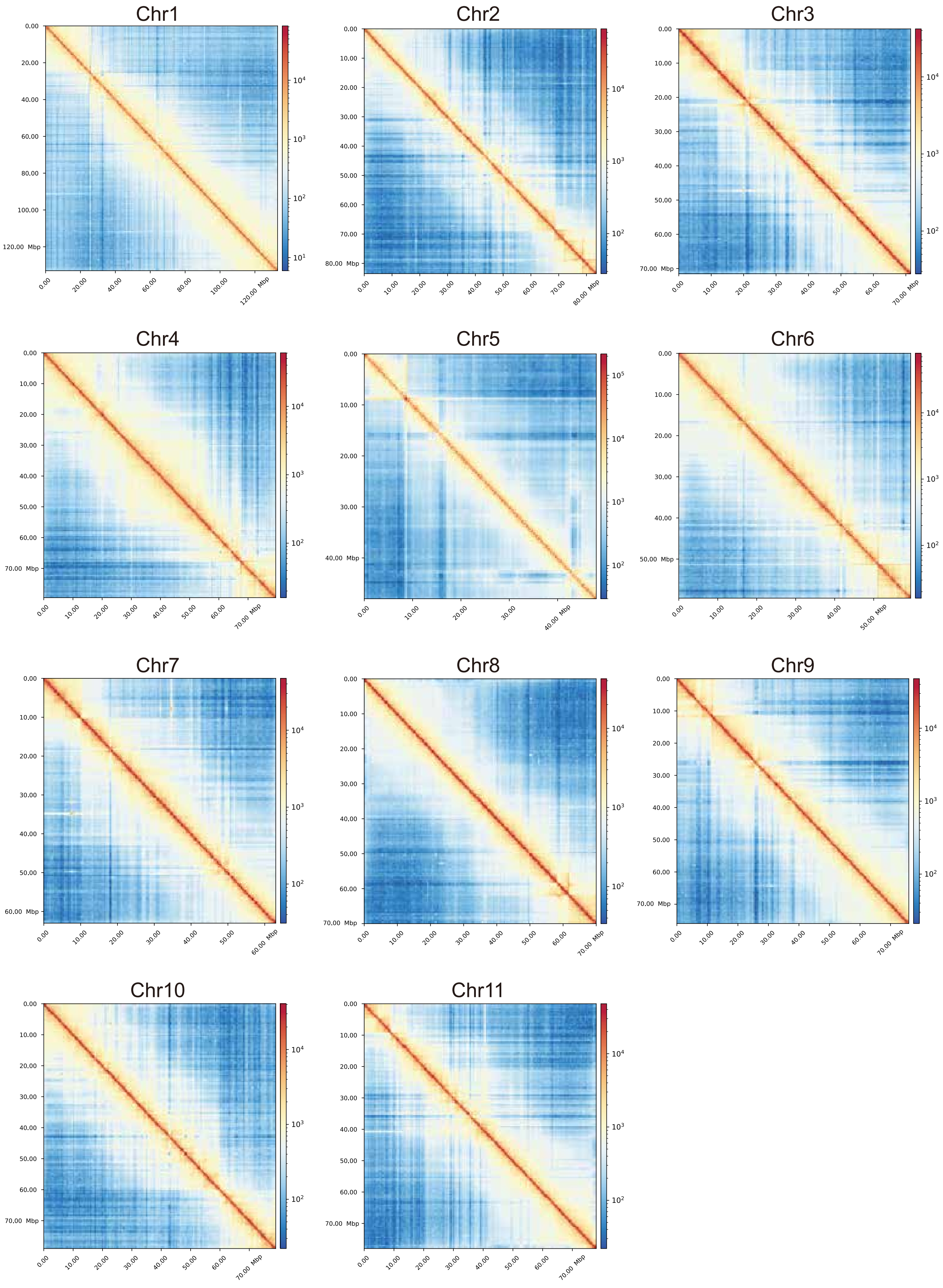

Figure S1. 11 pseudomolecules scaffolding based on chromosome conformation captured by Hi-C sequencing.

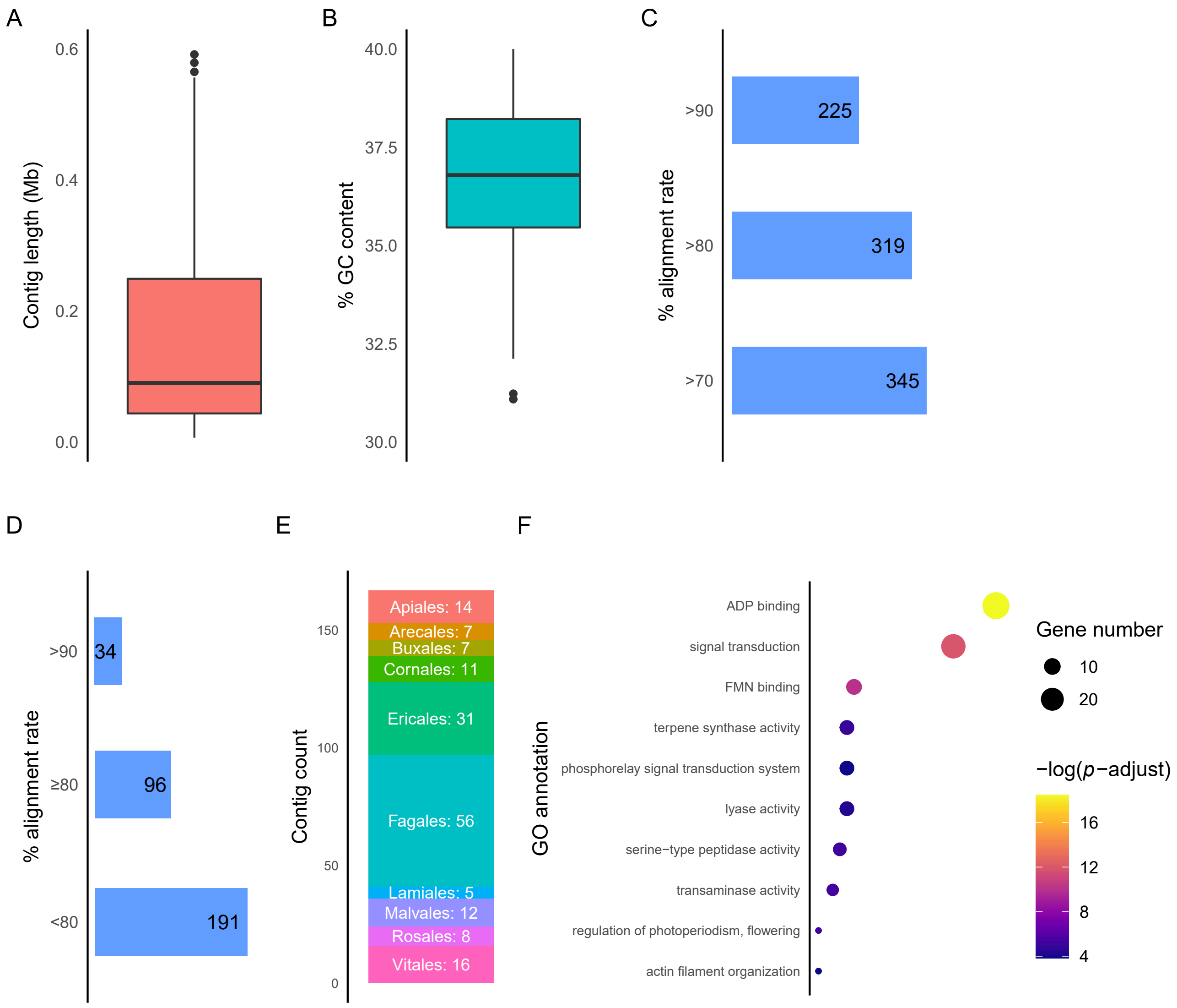

Figure S2. Overview for 345 unanchored contigs in *C. wilsoniana*. (A) Sequence length statistics of unanchored contigs. **(B)** GC content statistics of unanchored contigs. **(C)** Alignment rate statistics of unanchored contigs to 11 chromosomes. **(D)** Alignment rate statistics of unanchored contigs to the NT library. **(E)** Taxonomic identification of unanchored contigs. **(F)** GO analysis results of unanchored contigs.

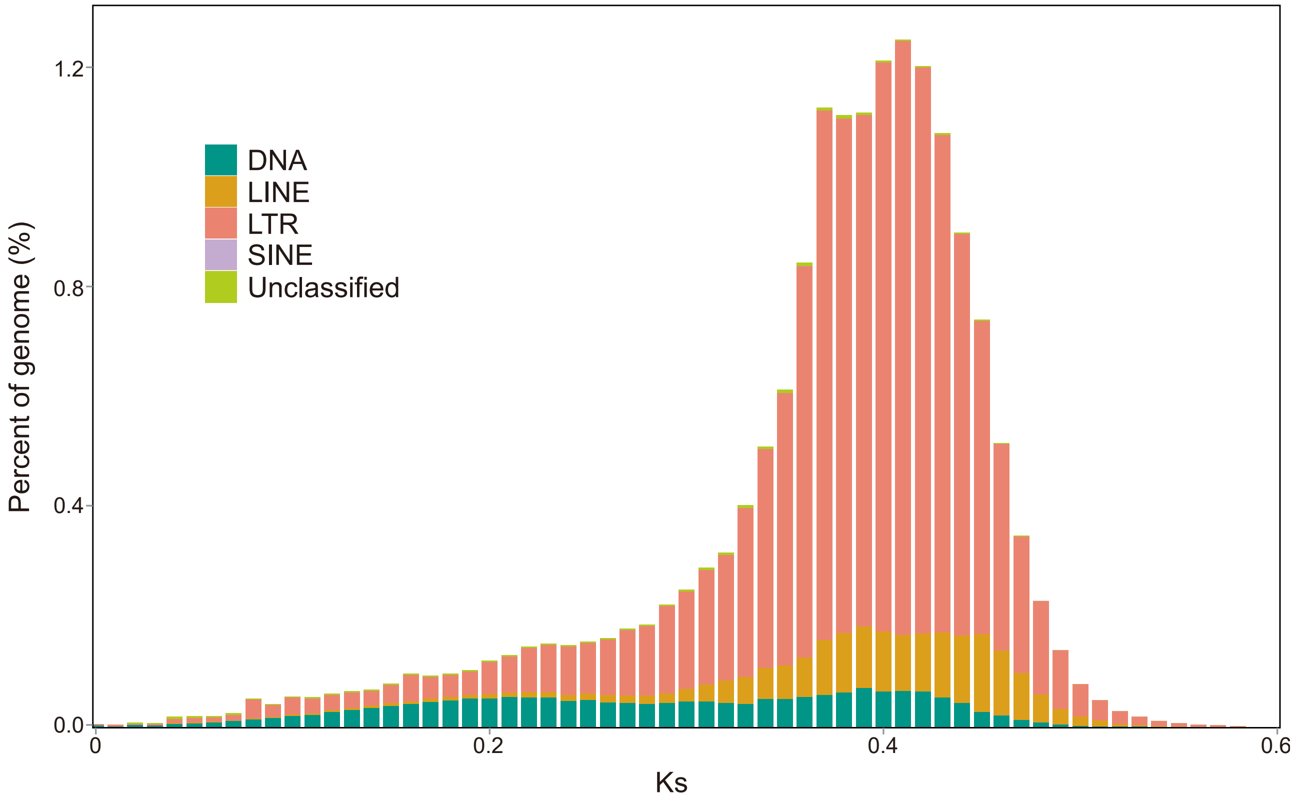

Figure S3. ivergence distribution of transposable elements in the genome of *C. wilsoniana*.

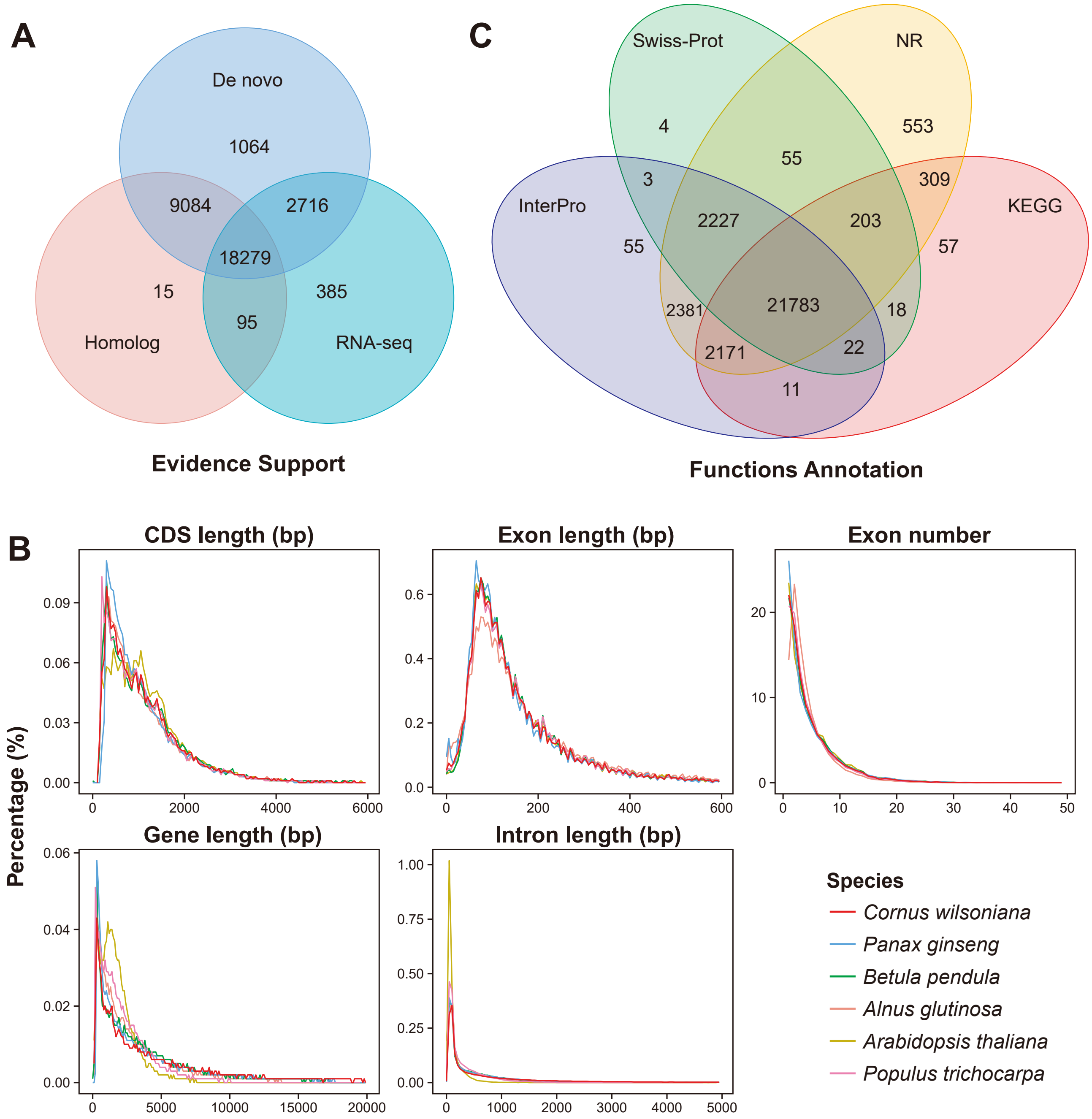

Figure S4. Gene structure and function annotation in the genome of *C. wilsoniana*. (A) The evidence supports for each gene set. Each evidence support is based on the principle that the gene sequence overlap is greater than 50%. **(B)** Comparison of gene structure of six relative species. The detailed data can be found in Table S9. **(C)** Statistical results of gene function annotation in four databases.

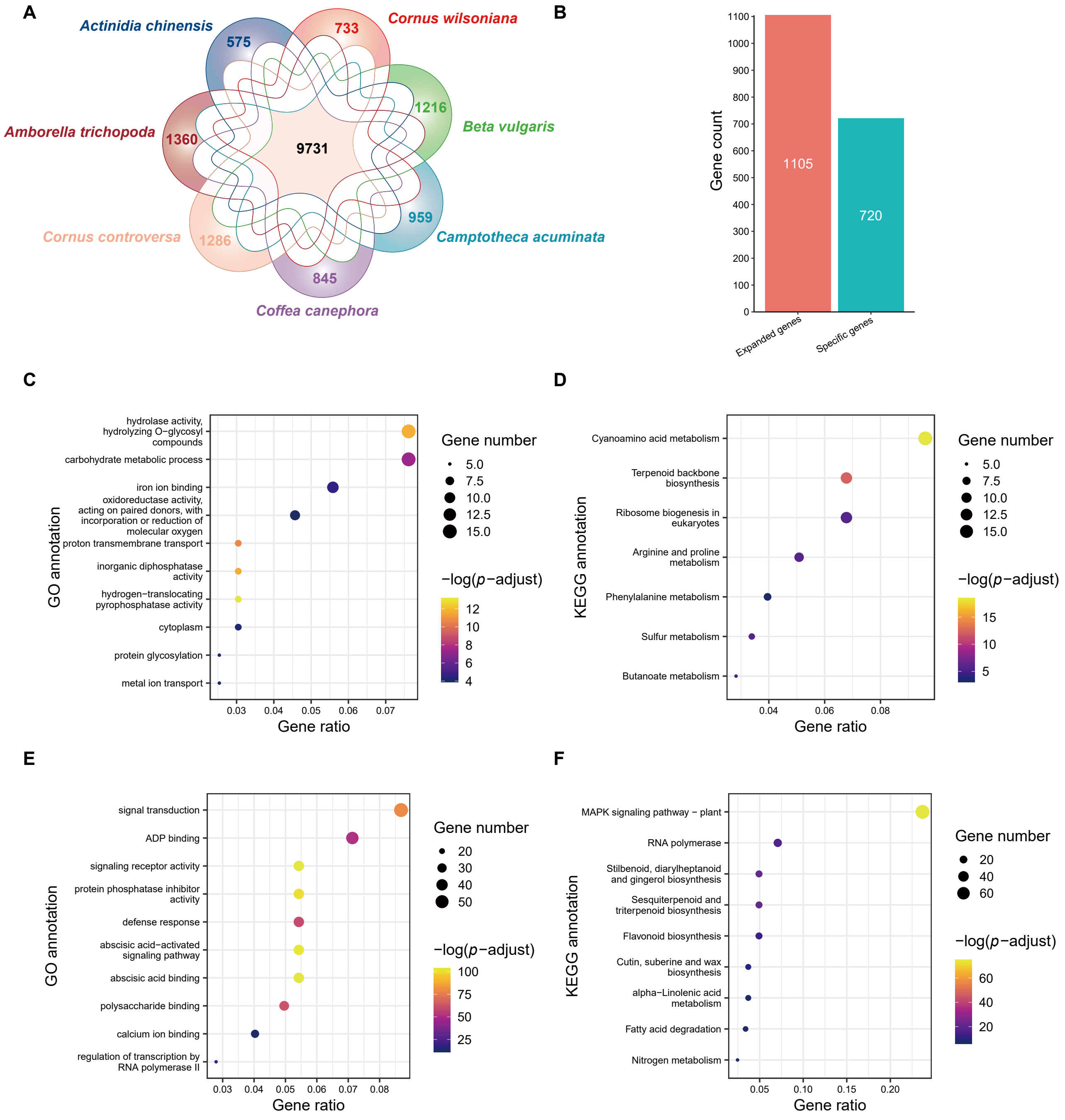

Figure S5. GO and KEGG annotation of *C. wilsoniana* expansion and specific families. (A) Venn diagram representing the clusters of gene families in *C. wilsoniana* and 6 related plants. **(B)** Statistics of expanded genes and specific genes quantities in *C. wilsoniana*. **(C)** GO annotation of *C. wilsoniana* expansion families. *p*-adjust value < 0.05. **(D)** KEGG pathway annotation of *C. wilsoniana* expansion families. *p*-adjust value < 0.05. **(E)** GO annotation of *C. wilsoniana* specific families. *p*-adjust value < 0.05. **(F)** KEGG pathway annotation of *C. wilsoniana* specific families. *p*-adjust value < 0.05.

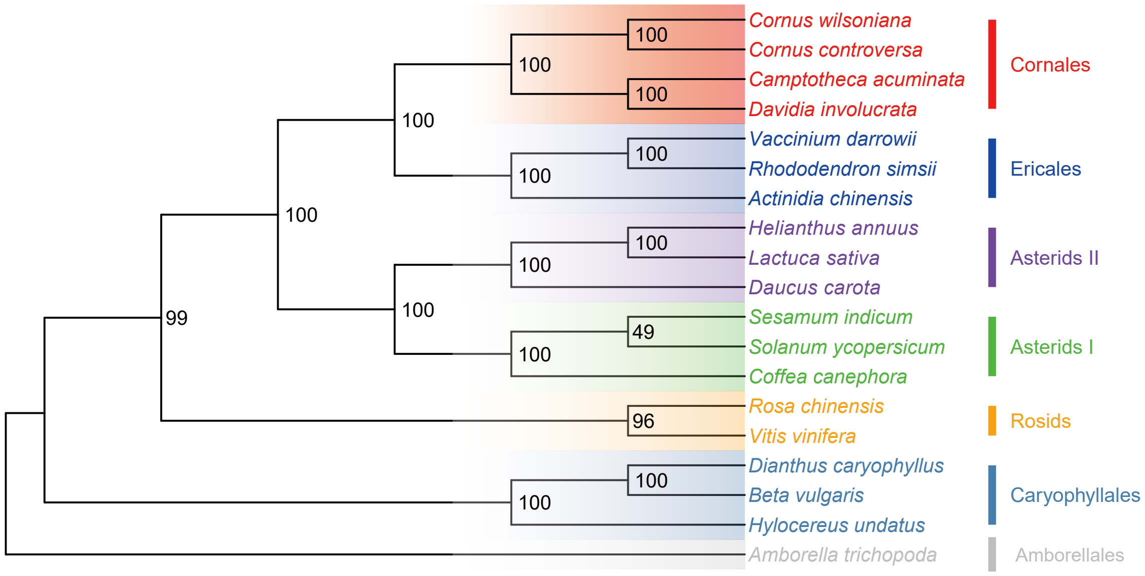

Figure S6. Phylogenetic trees of 19 species.

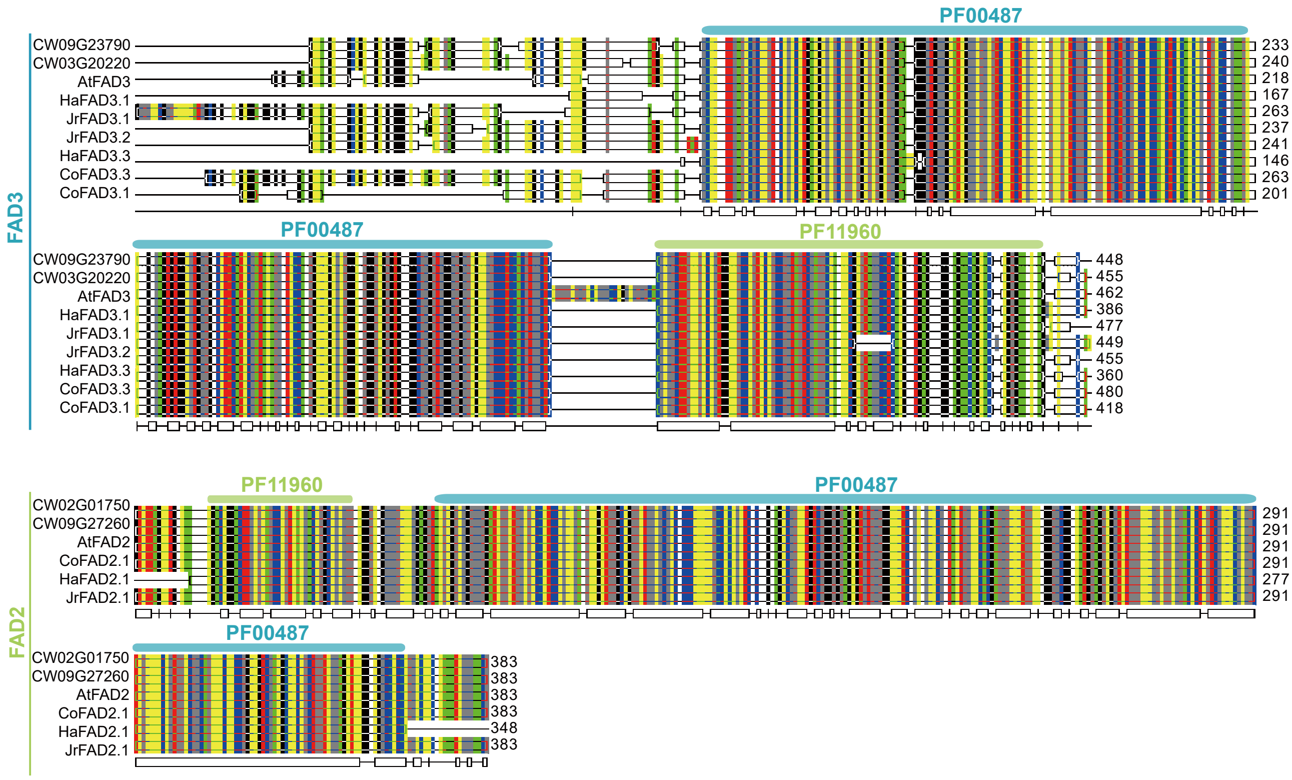

Figure S7. FAD protein sequence structures and domain (PF00487 and PF11960) locations.

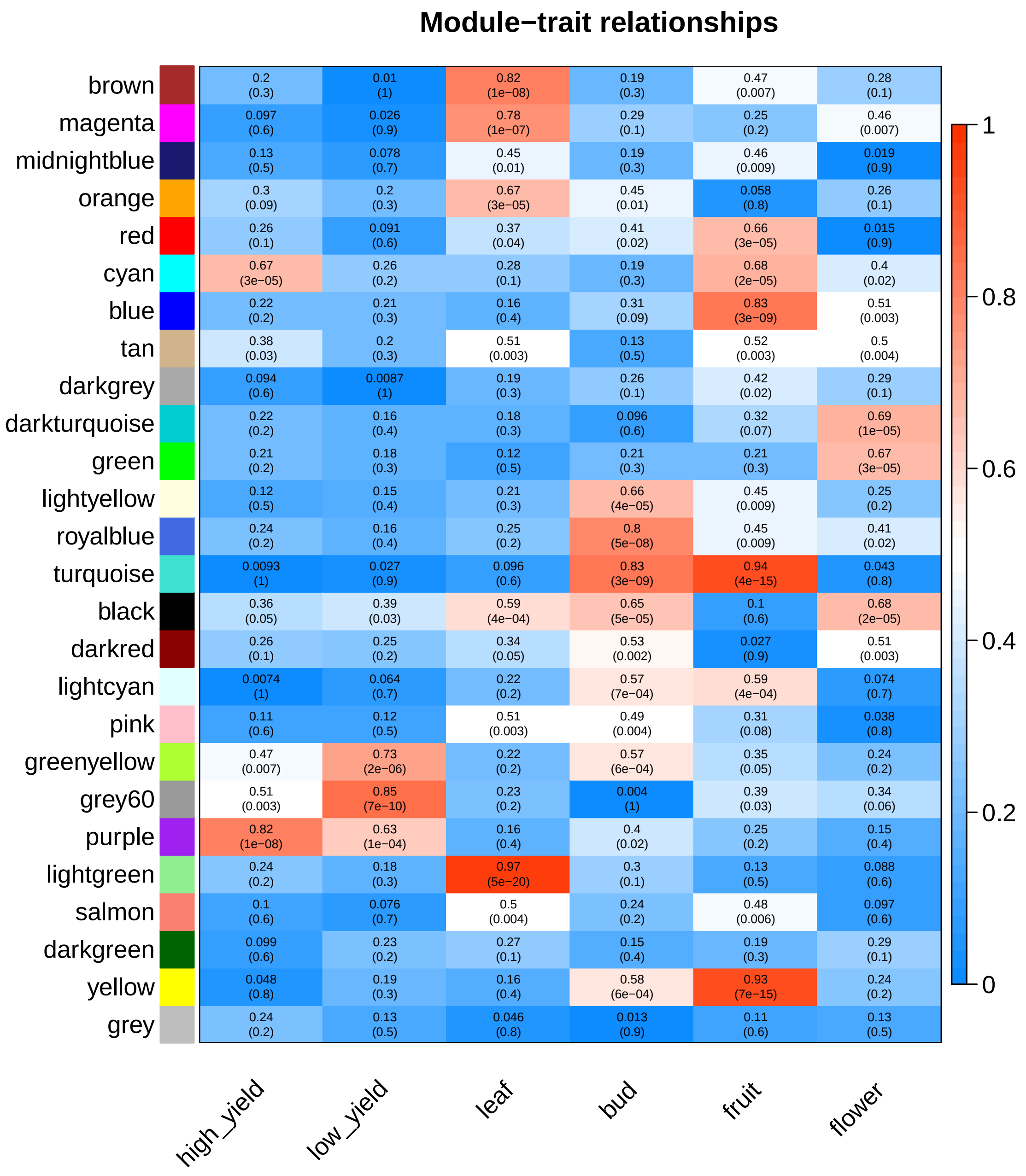

Figure S8. Module and trait relationships analysis by WGCNA.

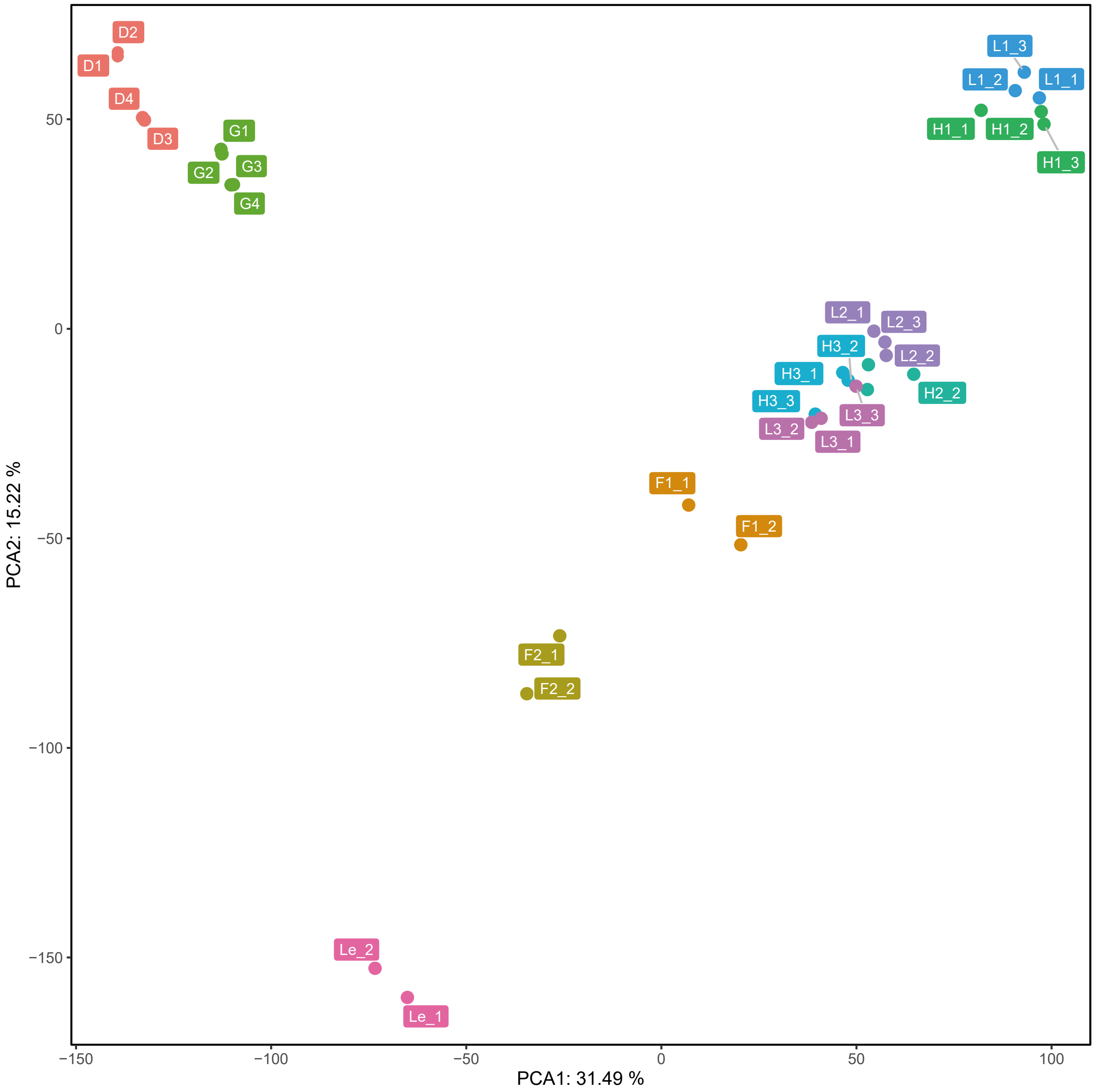

Figure S9. The PCA score plot of genes expression profile of the floral buds of high-yield and low-yield, flower, leaf and fruit.

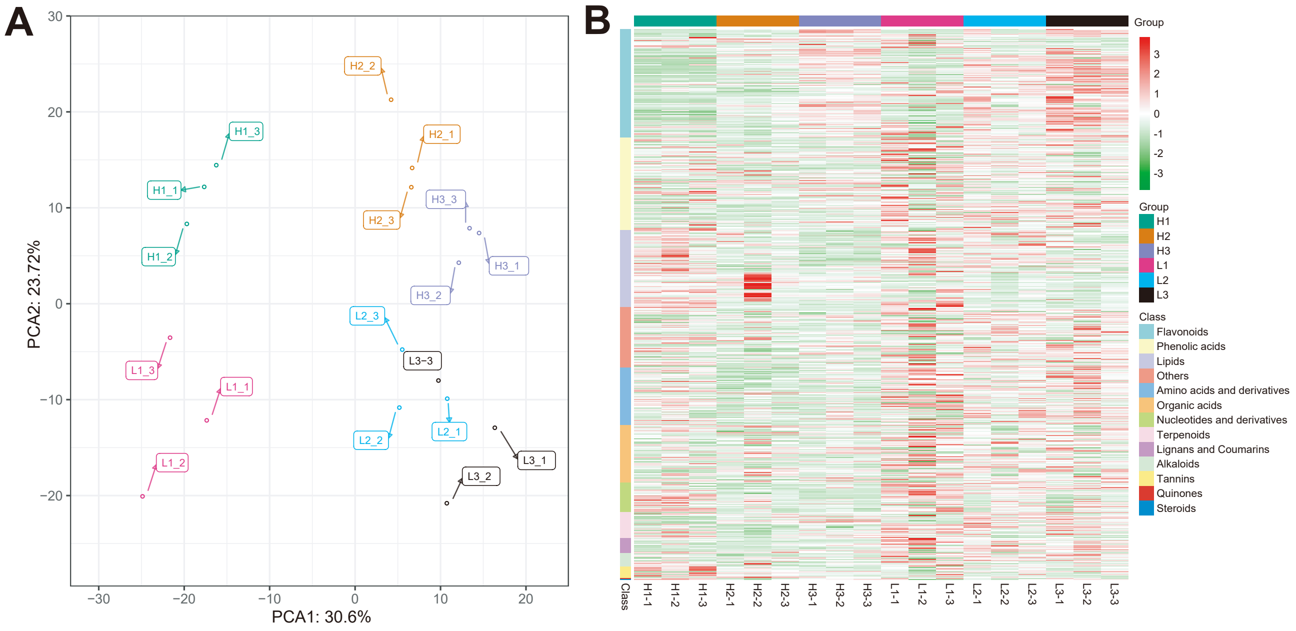

Figure S10. The PCA score plot (A) and heatmap (B) of metabolites expression profile in floral buds.

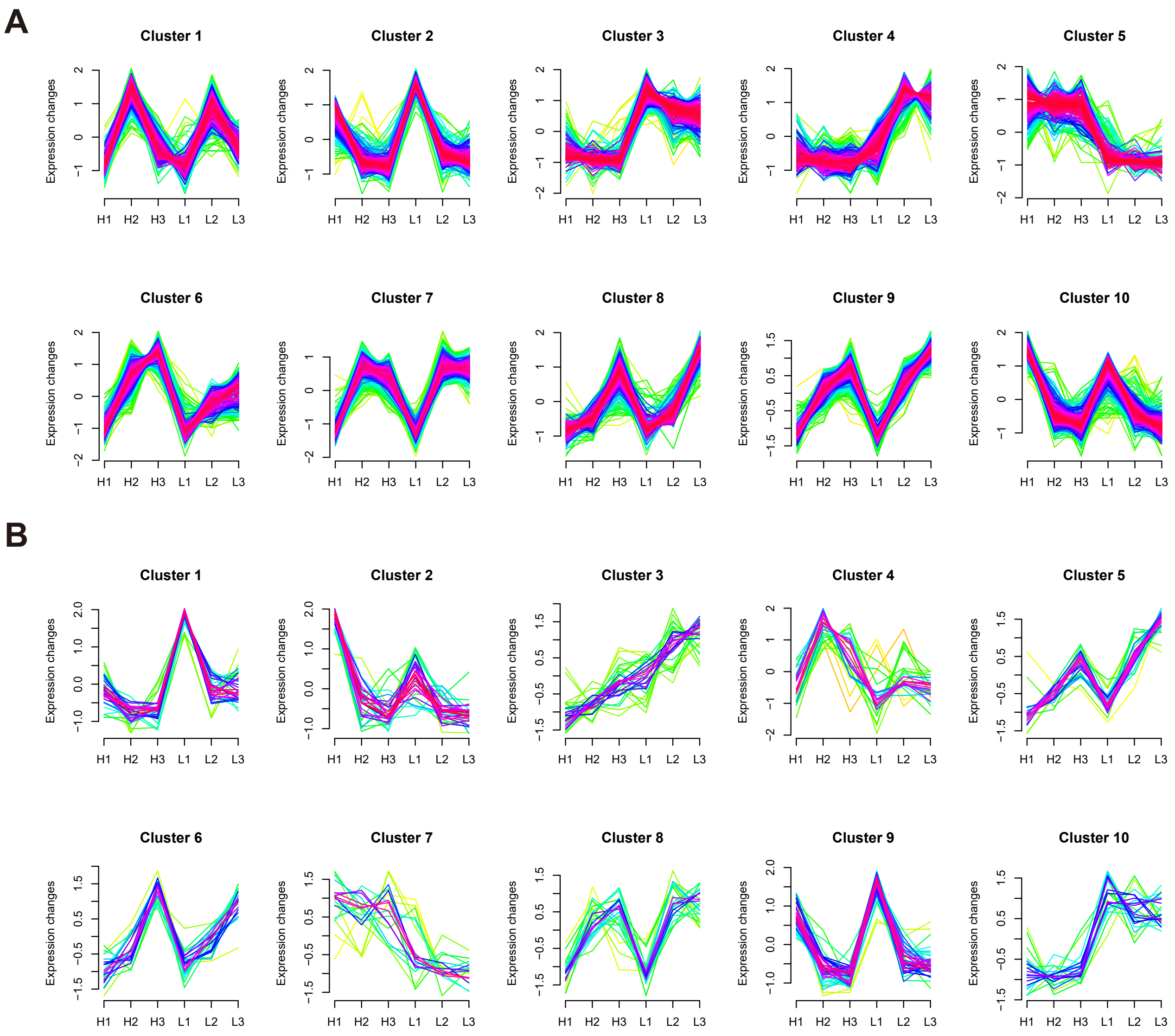

Figure S11. Trend analysis for DEGs and DEMs. (A) Trend analysis for DEGs. **(B)** Trend analysis for DEMs.

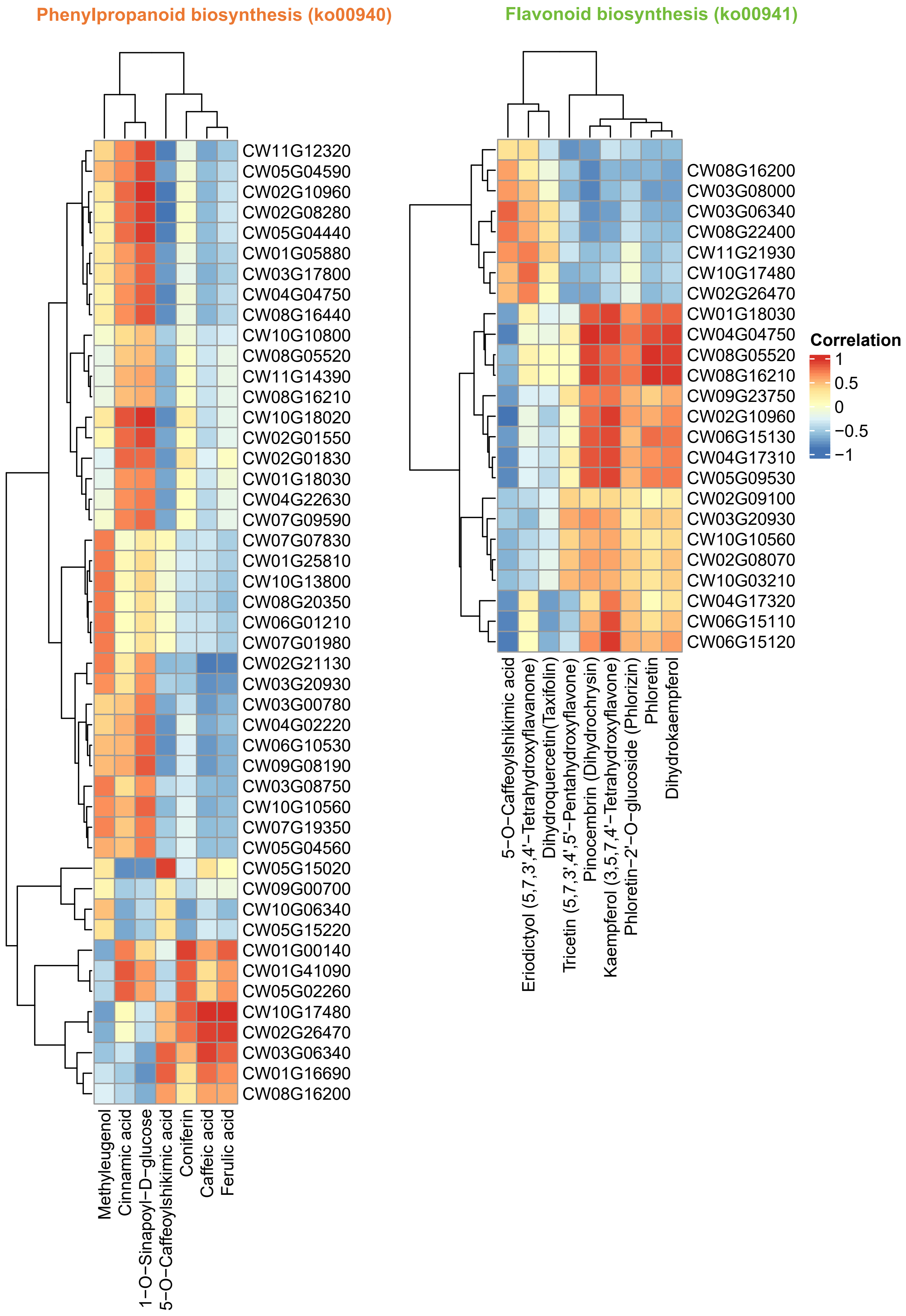

Figure S12. Correlation heatmap between DEGs and DEMs in phenylpropanoid and flavonoid biosynthesis.
